# Strategies to Formulate Value-Added Pastry Products from Composite Flours Based on Spelt Flour and Grape Pomace Powder

**DOI:** 10.3390/foods12173239

**Published:** 2023-08-28

**Authors:** Mariana-Atena Poiana, Ersilia Alexa, Isidora Radulov, Diana-Nicoleta Raba, Ileana Cocan, Monica Negrea, Corina Dana Misca, Christine Dragomir, Sylvestre Dossa, Gabriel Suster

**Affiliations:** 1 Faculty of Food Engineering, University of Life Sciences “King Michael I” from Timisoara, Aradului Street No 119, 300645 Timisoara, Romania; marianapoiana@usvt.ro (M.-A.P.); ileanacocan@usvt.ro (I.C.); monicanegrea@usvt.ro (M.N.); corinamisca@usvt.ro (C.D.M.); christine.dragomir98@gmail.com (C.D.); sylvestredossa04@gmail.com (S.D.); 2 Faculty of Agriculture, University of Life Sciences “King Michael I” from Timisoara, Aradului Street No 119, 300645 Timisoara, Romania; isidora_radulov@usvt.ro; 3 Faculty of Tourism and Rural Management, University of Life Sciences “King Michael I” from Timisoara, Aradului Street No 119, 300645 Timisoara, Romania; diana.raba@usvt.ro (D.-N.R.); gabrielsuster@usvt.ro (G.S.)

**Keywords:** spelt flour, grape pomace, composite flour, pastry products, phytochemical compounds, antioxidant properties

## Abstract

In recent years, sustainability has promoted new research to develop reformulation strategies for value-added food products by exploiting grape pomace. Grape pomace powder (GP) was used to substitute spelt flour (SF) at 0, 5, 10, 15, 20 and 25% to obtain three types of fortified pastry products: biscuits and cakes involving a chemical leavening agent, and rolls leavened by yeast. Proximate composition, total phenolic content (TPC), total flavonoids content (TFC), 1,1-diphenyl-2-picrylhydrazyl (DPPH) radical scavenging activity and ferric-reducing antioxidant power (FRAP) along with physical characteristics and sensory analysis of the enriched products were considered. The retention rate of the functional attributes of formulations in response to baking was also evaluated. Significant improvements in TPC, TFC and both antioxidant tests were achieved in the fortified products by the incremental incorporation of GP. With a substitution of 25% SF by GP, the following increases were recorded in biscuits, cakes and rolls over the control samples: 7.198-, 7.733- and 8.117-fold for TPC; 8.414-, 7.000- and 8.661-fold for TFC; 16.334-, 17.915- and 18.659-fold for FRAP and 16.384-, 17.908- and 18.775-fold for DPPH. The retention rates of TPC, TFC, FRAP and DPPH relative to the corresponding dough were 41–63%, 37–65%, 48–70% and 45–70%. The formulas leavened by yeast revealed higher functionality than those produced with a chemical raising agent. With the increase in GP, the elasticity and porosity gradually decreased for cakes and rolls, while the spread ratio of biscuits increased. Regarding sensory evaluation, all formulations with incorporated GP up to 10% were rated at an extremely pleasant acceptability level. The solutions derived from this study have great practical applicability for the development of new pastry formulations with improved functionality from GP valorisation.

## 1. Introduction

Fortification with functional ingredients derived from agro-food by-products is a valuable strategy for enhancing the nutritional properties of foods, well aligned with the growing interest in obtaining foods with health-promoting properties [1,2]. In line with circular economy concepts, there is now a strong focus on zero-waste technologies, with by-products being effectively reintegrated into the food chain as a source of biologically active species to produce functional products [3,4,5]. Wine making leads to the generation of huge quantities of grape pomace—around 7–9 million tonnes/year worldwide [6]. Grape pomace represents a phenolic-rich matrix with multiple benefits to human health, since after the winemaking, about 70% of the phenolic fraction from grapes, consisting mainly of tannins, phenolic acids, anthocyanins and resveratrol, remains in the waste [7,8,9]. Significant efforts have been made in recent years to prove the functional potential of grape pomace and to explore its use as a value-added food ingredient for the development of new foods for the prevention of nutrition-related diseases [10,11,12]. Grape pomace valorisation is important both ecologically and as a sustainable source of high-quality bioactive compounds with significant antioxidant activity [13,14]. Its incorporation into cosmetic, food or pharmaceutical products represents a market opportunity for wine producers and a strategy for increasing the dietary intake of phenolic compounds [15,16].

A number of food products have been successfully enriched with phenolic compounds by incorporating grape pomace, either as natural extracts or as powders from seeds, skins, or whole grape marc. Bakery products represent the category with the most applications of grape pomace, but there are currently no commercial products on the market that contain grape pomace as a substitute for conventional wheat flour [17,18].

Since flour products are consumed all over the world, they can be an excellent basis for supplementation with bioactive-rich materials [19,20]. Improving the functionality of these products remains of great interest, even though it has been reported that additions of more than 15% may negatively affect the taste of the developed products [18].

Grape pomace has been included in various bakery products as a partial replacement for wheat flour to decrease the output gluten and increase elasticity and dietary fibre level [21], as follows: grape seed flour was incorporated in cereal bars, pancakes, noodles [22], butter biscuits [23] and bread [24], and grape pomace was incorporated into bread [20,21,25], biscuits [26], crackers [27], pasta [19,28,29], cakes [30] and cookies [31,32], while grape skin powder has been included in pasta recipes [33]. The incorporation of grape pomace could be used as a feasible way to develop new pastry formulations for the following reasons: it is an alternative source of high-value dietary fibre and phenolic compounds; it is a substitute for modified food starch; and it results in products with reduced gluten content and an increase in bioactive compounds [34].

Although there are numerous studies on the changes induced in the chemical, technological and sensory properties of bakery and pastry products through the addition of grape pomace, the type of flour used has not always been mentioned, nor the grape variety from which the marc originated, or whether it was included in whole or fractionated form or whether the basic flour was composed solely of wheat flour [19,35]. As such, the lack of standardisation of studies is a major factor that affects the comparison of the properties of the products formulated by including grape pomace in the recipe [19]. Today, the milling and bakery industry is increasingly focused on developing nutritionally and functionally high-value products based on sustainable and locally accessible resources that meet consumer demands for a healthy diet. Composite flours, defined as mixtures of powdered ingredients consisting of cereal flours or milling products combined with powders from fruit, vegetables and food by-products of plant origin, have become increasingly popular in the pastry industry due to their nutritional and functional value, with the advantage of being used immediately, without prior preparation, facilitating and speeding up the manufacturing process [36].

The interest in spelt wheat (*Triticum spelta* L.) has increased in the last few years, especially for organically grown wheat [37,38]. This is mainly due to the superior nutritional composition of spelt wheat compared to common wheat flour, reflected by a higher concentration of mineral nutrients, fibre, protein, lipids, antioxidant properties and phenolic compounds [39,40]. There are no reported studies on the impact of fortifying pastry products obtained from spelt flour with phenolic compounds provided by grape pomace on their nutritional, bioactive and sensory attributes.

This study investigated the partial replacement of spelt flour with grape pomace powder in the recipe of three types of pastry products, such as biscuits and cakes where a chemical raising agent was involved, and rolls leavened by yeast. In this respect, some important operational issues are addressed: What impact was achieved by increasing the level of GP on the nutritional, bioactive and sensory properties of the pastry formulas? How does the baking process influence the rate of retention of functional properties of products in relation to the corresponding dough? Are there differences in the bioactive compound content and antioxidant properties of pastry products depending on the production process, and which of the three types of pastry promotes the highest level of functional properties? What is the recommended percentage of GP to be incorporated as the partial replacement of spelt flour without affecting the sensory attributes of pastry formulas? To answer these questions, an integrative study was carried out starting from the proximate composition of spelt flour, grape pomace powder and pastry products, followed by investigations into the content of total phenolic compounds, total flavonoids, ferric-reducing antioxidant power and DPPH radical scavenging activity in the products and corresponding dough, as well as the sensory evaluation of pastry formulas.

## 2. Materials and Methods

### 2.1. Chemicals and Reagents

Folin–Ciocalteu reagent, ferric chloride hexahydrate and sodium carbonate anhydrous were purchased from Merck (Darmstadt, Germany); standards of gallic acid and quercetin, 6-hydroxy-2,5,7,8-tetramethylchroman-2-carboxylic acid (Trolox), 1,1-diphenyl-2-picrylhydrazyl (DPPH), 2,4,6-tris(2-pyridyl)-*s*-triazine (TPTZ), ferrous sulphate heptahydrate, sodium acetate anhydrous, glacial acetic acid, hydrochloric acid 0.1 M, aluminium nitrate nonahydrate and sodium nitrite were purchased from Sigma-Aldrich (Taufkirchen, Germany); ethanol 96% was acquired from Chimreactiv (Bucharest, Romania). All reagents were of analytical grade.

### 2.2. Ingredients and Manufacture of Pastry Products

The whole spelt wheat (*Triticum aestivum* ssp. *Spelta*) flour (SF) was purchased from SC PRONAT SRL, Sanandrei, Timis County, Romania. 

Red grapes (*Vitis vinifera* L., cultivar Merlot) were provided from Recas vineyard, (Timis County, Romania, vintage year 2021). Raw grape pomace was obtained following a laboratory-scale vinification process, after a maceration phase of 10 days and pressing. The raw material was dried at 60 °C for a total period of 24 h, 8 h per day for 3 days in a row, in a Binder convective oven (Binder GmbH, Tuttlingen, Germany) until the moisture content was less than 5% to ensure microbial safety [41,42]. After cooling at 20 °C, the dried grape pomace was milled with a Grindomix GM 200 cutting mill (Retsch GmbH, Haan, Germany), and then the material was sieved with a 60-mesh sieve and the grape pomace powder (GP) was vacuum-packed in polypropylene bags and stored at room temperature in the dark until analysis or until the composite flours were obtained, as described by Tolve et al. [25]. The microbiological analysis of GP performed by standard methods for counting total aerobic mesophilic germ count (ISO 4833:2003) [43], *Enterobacteriaceae* count (ISO 21528-2:2004) [44], yeast and mould count (ISO 21527:2008) [45] and the presumptive number of *Bacillus cereus* germs (SR EN ISO 21871:2006) [46] revealed the following results: total aerobic mesophilic germ count: 2 × 10^2^ colony-forming units (CFU)/g, *Enterobacteriaceae* count: 0 CFU/g, yeasts and mould count: 4 × 10 CFU/g and *Bacillus* cereus count: 1 × 10^2^ CFU/g. Considering the low moisture of the sample, GP is protected against microbial development. Legislation does not present microbiological limits for GP and this is the reason for referring to the legislative values for flours used in baking, especially as GP will be incorporated into the recipe of pastry products. GP showed low microbial counts, below the maximum level allowed by Regulation (EC) No. 2073/2005 [47], and is compatible with its further use when also considering that the manufacture of these products involves high-temperature treatment.

Five composite flours were prepared by replacing SF with GP in the proportions of 5, 10, 15, 20 and 25% (*w*/*w*) and labelled as SF95GP5 (95% SF + 5% GP), SF90GP10 (90% SF + 10% GP), SF85GP15 (85% SF + 15% GP), SF80GP20 (80% SF + 20% GP) and SF75GP25 (75% SF + 25% GP). The composite flour was used in the production of three types of enriched pastries that differ in the type of dough, such as biscuits, cakes and rolls, in the pilot bakery unit of the University of Life Sciences “King Michael I”, Timisoara. Five fortified formulations and one control (100% SF) were developed for each type of pastry according to the recipes shown in Table 1 and were labelled as follows: BSF, BSF95GP5, BSF90GP10, BSF85GP15, BSF80GP20 and BSF75GP25 for biscuits, CSF, CSF95GP5, CSF90GP10, CSF85GP15, CSF80GP20 and CSF75GP25 for cakes, and RSF, RSF95GP5, RSF90GP10, RSF85GP15, RSF80GP20 and RSF75GP25 for rolls.

The ingredients involved in the production recipes such as sugar (Margaritar, Agrana Romania S.R.L., Romania), milk with 3.5% fat (ProdLacta, SC PRODLACTA SA, Brasov, Romania), butter with 80% fat (ProdLacta, SC PRODLACTA SA, Brasov, Romania), eggs (Agricola, Bacau, Romania), baking powder (Dr. Oetker Original Backin, Dr. Oetker SRL, Arges County, Romania), salt (Salrom, Bucuresti, Romania) and fresh yeast (Pakmaya, ROMPAK SRL, Pascani, Romania) were purchased from a local supermarket. Baking powder was used as a chemical raising agent for the biscuits and cakes, while yeast (*Saccharomyces cerevisiae*) was used as a natural leavening agent to obtain the rolls.

**Figure 1:**
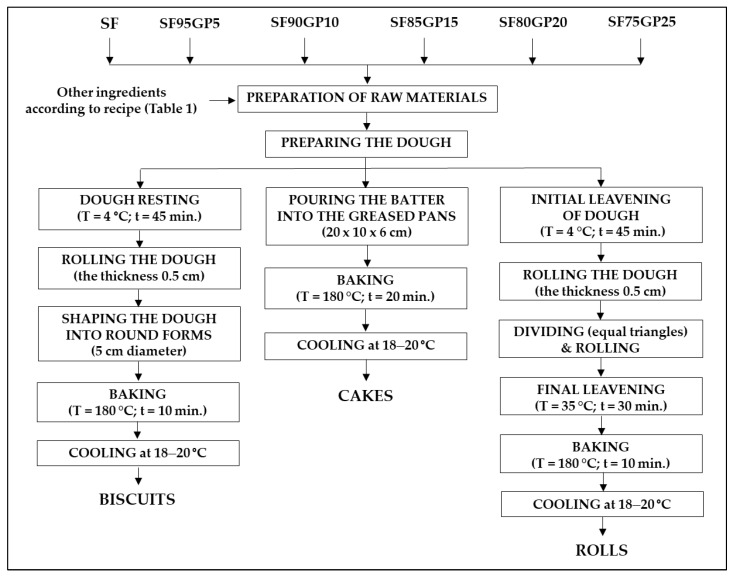
Technological flowchart for production of pastry products. SF: spelt flour; GP: grape pomace; SF95GP5: 95% spelt flour + 5% grape pomace; SF90GP10: 90% spelt flour + 10% grape pomace; SF85GP15: 85% spelt flour + 15% grape pomace; SF80GP20: 80% spelt flour + 20% grape pomace; SF75GP25: 75% spelt flour + 25% grape pomace.

Control samples of each type of product were prepared according to traditional methods, applied on a small-scale level. The recipes were previously tested in the bakery and pastry processing unit of the Didactic and Experimental Station of the University of Life Sciences “King Michael I”, Timisoara. The same methods were used for the pastry formulas with GP incorporation, except that the SF was replaced by composite flours. The production process of the pastry products is shown in Figure 1.

**Figure 2:**
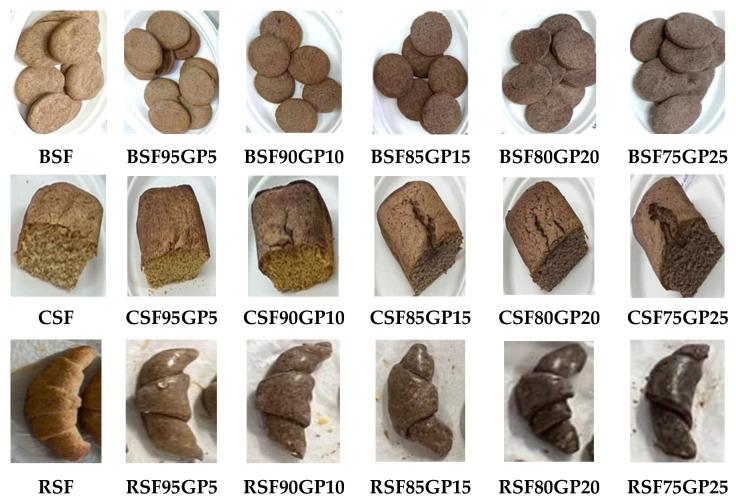
Pastry products after baking. BSF, CSF, RSF (control: biscuits, cakes and rolls); BSF95GP5, CSF95GP5, RSF95GP5, (biscuits, cakes and rolls: 95% spelt flour + 5% grape pomace); BSF90GP10, CSF90GP10, RSF90GP10 (biscuits, cakes and rolls: 90% spelt flour + 10% grape pomace); BSF85GP15, CSF85GP15, RSF85GP15 (biscuits, cakes and rolls: 85% spelt flour + 15% grape pomace); BSF80GP20, CSF80GP20, RSF80GP20 (biscuits, cakes and rolls: 80% spelt flour + 20% grape pomace); BSF75GP25, CSF75GP25, RSF75GP25 (biscuits, cakes and rolls: 75% spelt flour + 25% grape pomace).

The dough for the biscuits and rolls was prepared by mixing the ingredients for 5 min with a planetary mixer (Moulinex, 800 W, Moulinex SA, Paris, France) a in a single-phase procedure. Before use, the fresh yeast was crumbled into a bowl, and then a tablespoon of sugar and a little lukewarm milk was added and stirred to dissolve before being left to stand for 15 min, during which time the mixture increased in volume and bubbles formed on the surface. The batter for the cakes was prepared using the following procedure: the egg whites were whipped for 5 min with salt using a hand mixer with five speeds (Bosch MFQ49300, 850 W, Robert Bosch GmbH, Stuttgart, Germany) at top speed. Then, the sugar was added gradually and mixed for 1 min at full speed. Separately, the egg yolks were mixed for 1 min at high speed with butter previously kept at room temperature for 1h and then this mixture was incorporated into the foam from egg whites. SF, or composite flours, depending on the formulation, was slowly added to the resulting mixture along with the baking powder. The dough obtained was labelled according to each pastry product: DBSF, DBSF95GP5, DBSF90GP10, DBSF85GP15, DBSF80GP20 and DBSF75GP25 for biscuits; DCSF, DCSF95GP5, DCSF90GP10, DCSF85GP15, DCSF80GP20 and DCSF75GP25 for cakes; and DRSF, DRSF95GP5, DRSF90GP10, DRSF85GP15, DRSF80GP20 and DRSF75GP25 for rolls. Baking of the pastries was carried out in an electric convection oven (Esmach, 1200 W, 50 Hz, Esmach Ali Group SRL, Grisignano Di Zocco, Italy) set at 180 °C. All pastry products showed a similar baking behaviour. Each pastry formula was produced in three batches on the same day. Samples were taken from each batch and dough formula, packed in polypropylene food storage bags, sealed and stored at −20 °C until chemical analysis. After baking and cooling the products at room temperature for 12 h, a representative sample for each batch and pastry formulation was made up from randomly selected subsamples, packed in polypropylene bags, sealed and stored at −20 °C until chemical analysis. The remaining pastry products were packaged in plastic food storage containers and distributed for sensory analysis. Figure 2 illustrates the baked pastries, control samples and the formulas with GP as an SF substitute.

### 2.3. Proximate Composition and Energy Value Evaluation of SF, GP and Pastry Products

The proximate composition of SF, GP and pastry products was evaluated in accordance with the standard method described by the Association of Official Analytical Chemists [48]. The carbohydrate content (%) was calculated by subtracting the protein, ash, lipid and moisture from 100. The energy value was calculated in accordance with Das et al. [49], taking into account that 1 g of carbohydrates provides 4 calories, 1 g of protein provides 4 calories and 1 g fat provides 9 calories, as shown in Equation (1).

Energy value (kcal/100 g) = carbohydrates (%) × 4 + lipids (%) × 9 + proteins (%) × 4(1)

### 2.4. Phytochemical Content and Antioxidant Activity of SF, GP and Pastry Products

#### 2.4.1. Alcoholic Extract Preparation

The total phenolic content, total flavonoid content and antioxidant activity were assessed in ethanol extracts following the procedure described by Litwinek et al. [50] with minor modifications. From each sample of SF, GP, pastry formulas and corresponding dough, 1 g was weighed into lidded containers and mixed with 10 mL of 70% (*v*/*v*) ethanol. The extraction was carried out for 120 min under continuous stirring at room temperature using an IDL magnetic stirrer (IDL GmbH & Co KG, Nidderau, Germany), and then the mixtures were centrifuged at 10,000 rpm for 10 min (Hettich EBA 21, Andreas Hettich GmbH & Co. KG., Tuttlingen Germany). The supernatant was carefully collected and the residue was further washed with 70% (*v*/*v*) ethanol and subjected to extraction under continuous stirring for a further 60 min at room temperature, and then centrifuged at 10,000 rpm for 10 min. Afterwards, the supernatants were mixed and stored in darkness at −20 °C until analysis. For each sample, the extraction was performed in triplicate and each replicate was subsequently used in the analysis.

#### 2.4.2. Evaluation of Total Phenolic Content 

The method applied to quantify the total phenolic content (TPC) was based on the oxidation of hydroxyl groups of phenolic compounds in alkaline media using Folin–Ciocalteu reagent, as described by Tolve et al. [25] and Blanch at al. [51]. Prior to analysis, the alcoholic extracts were diluted with distilled water, as follows: 1:2.5 (*v*/*v*) for SF, pastry products and dough, and 1:50 (*v*/*v*) for GP. Further, 0.5 mL of diluted extracts were mixed with 2.5 mL of Folin–Ciocalteu reagent previously diluted 1:10 (*v*/*v*) with distilled water. Next, 2 mL of 7.5% Na_2_CO_3_ solution was dosed and the obtained mixture was allowed to incubate for 30 min at 50 °C in the INB500 thermostat (Memmert GmbH, Schwabach, Germany), after which the absorbance was recorded at a wavelength of 750 nm using the Specord 205 UV–Vis spectrophotometer from Analytik Jena Inc. (Jena, Germany) versus a blank sample prepared under the same experimental conditions. TPC was calculated based on a calibration curve plotted by using standard gallic acid solutions with concentrations ranging from 0.1 to 1.0 µM gallic acid equivalents (GAE)/mL. TPC was reported as mg GAE/100 g dry weight (DW) of sample. 

#### 2.4.3. Evaluation of Total Flavonoids Content (TFC)

The total flavonoids content (TFC) of the samples was assessed according to the procedure described by Al-Farsi et al. [52] with minor modifications. Briefly, a 3.0 mL aliquot of ethanol extract was mixed with 4.5 mL of distilled water and 1 mL of 0.3% NaNO_2_ solution. The mixture was allowed to incubate for 6 min at 20 °C, and then 1 mL of 10% Al(NO_3_)_3_ was dosed and after another 6 min, 10 mL of 4% (*w*/*w*) NaOH solution was added. The volume of the mixture was made up to 25 mL with 70% (*v*/*v*) ethanol and the absorbance was read at 510 nm after 15 min, against a blank sample of 70% (*v*/*v*) ethanol. The calibration curve was plotted using standard quercetin (QE) solutions in the concentration range 0.5–50 μg/mL. TFC was reported as mg QE/100 g DW of sample.

#### 2.4.4. Assessment of Antioxidant Activity by 1,1-Diphenyl-2-picrylhydrazyl (DPPH) Assay

The free radical scavenging activity of the samples was evaluated by DPPH assay [3], using a 0.1 mM DPPH solution prepared in 70% (*v*/*v*) ethanol. This method is frequently used to evaluate the antioxidant activity of the samples under investigation based on their ability to scavenge free radicals. The ethanol extracts previously obtained were further diluted with 70% (*v*/*v*) ethanol at a ratio of 1:5 (*v*/*v*) for SF, pastry products and dough, respectively, and 1:100 (*v*/*v*) for GP. Next, a 1.0 mL aliquot of the diluted extracts was mixed with 2.5 mL of 0.1 mM DPPH solution in 70% (*v*/*v*) ethanol. The mixtures were homogenised using a hot plate stirrer (IDL, IDL GmbH & Co KG, Nidderau, Germany), and incubated for 30 min in the dark, at a temperature of 20 °C. The absorbance of the mixture was read at a wavelength of 517 nm versus 70% (*v*/*v*) ethanol. Under the same working conditions, a control sample was also prepared, consisting of a mixture of 1 mL of 70% (*v*/*v*) ethanol and 2.5 mL of 0.1 mM DPPH solution in 70% (*v*/*v*) ethanol. Equation (2) was used to compute the DPPH radical scavenging activity:

(2) $$\mathrm{DPPH}\;\mathrm{Scavenging}\;\mathrm{Activity}\;(\%)=\frac{A_{c}-A_{s}}{A_{c}}\times 100$$

where A_c_ represents the absorbance of the control sample and A_s_ represents the absorbance read in the presence of the test sample. The calibration curve DPPH scavenging activity (%) versus Trolox concentration (µg/mL) was plotted using standard solutions of Trolox with concentrations in the range 1.0–25 µg Trolox/mL [53]. Based on the calibration curve, in the first step, the Trolox equivalent (TE) concentration was calculated. Then, taking into account the molar mass of Trolox and the concentration of the sample solution in g/mL, the antioxidant activity was determined and expressed in µM Trolox equivalent (TE)/g DW of sample.

#### 2.4.5. Assessment of Antioxidant Activity by Ferric-Reducing Antioxidant Power (FRAP) Assay

The total antioxidant potential of the samples was assessed based on the ferric reducing antioxidant power (FRAP) assay based on the ability of antioxidant compounds from ethanol extracts to reduce Fe^3+^ from colourless ferric complex (Fe^3+^–tripyridyltriazine) to Fe^2+^ due to the action of electron-donating antioxidant species at a low pH [54]. The deep blue-coloured Fe^2+^–tripyridyltriazine complex shows a maximum absorbance at 593 nm [54]. Basically, the working solution was prepared from 100 mL acetate buffer (pH = 3.6), 10 mL of 10 mM TPTZ solution in 40 mM HCl and 10 mL of 20 mM FeCl_3_ · 6H_2_O solution. Prior to analysis, the extracts were diluted with distilled water, as follows: 1:2.5 (*v*/*v*) for SF, 1:50 (*v*/*v*) for GP and 1:2.5 (*v*/*v*) for pastries and dough. Then, 0.5 mL of diluted extracts were left to react with 2.5 mL of working solution at a temperature of 37 °C for 30 min, and then the absorbance was read at a wavelength of 593 nm versus a blank sample obtained under the same operational conditions. The results were calculated as µM Fe^2+^ equivalent/g DW of sample based on a calibration curve plotted using FeSO_4_ · 7H_2_O solutions with concentrations ranging from 0.05 to 0.5 µM Fe^2+^ equivalents/mL.

### 2.5. Assessment of Physical Characteristics 

The cake and roll formulas were subjected to porosity and core elasticity assessment according to SR 91:2007 [55]. For biscuits, the spread ratio (SR) was determined as the ratio of biscuit diameter to biscuit height [56].

### 2.6. Sensory Evaluation 

The sensory analysis was carried out by a panel consisting of 27 assessors (12 males and 15 females) recruited from the staff and students at University of Life Sciences “King Michael I”, Timisoara (Romania), non-smokers, aged between 20 and 50, regular consumers of bakery products, without known cases of food allergies or food intolerances. Participation in this study was voluntary. The panellists were trained prior to the analysis to identify the attributes to be evaluated. The sensory analysis of the products followed laboratory ethical guidelines and written informed consent was obtained from each evaluator in conformity with the European Union guidelines on Ethics and Food-Related Research [57]. The samples were presented in cardboard plates with three-digit characters, one at a time to each evaluator. The panellists were asked to rinse their mouths with still water between sample evaluations. The sensory characteristics (appearance, flavour, texture, taste and overall acceptability) of the coded samples were assessed based on their liking degree using a five-point hedonic scale from 1—dislike extremely to 5—like extremely. The score ranges and level of acceptability were grouped as follows: 1.00–1.49 (extremely unpleasant); 1.5–2.49 (slightly unpleasant); 2.50–3.49 (neither pleasant nor unpleasant); 3.5–4.49 (slightly pleasant); 4.5–5.00 (extremely pleasant) [58].

### 2.7. Statistical Analysis

The data were obtained from three independently performed experiments, each of which were analysed in three replicates. The results represent an average of three independent experiments and were expressed as mean ± standard deviation (SD). One-way analysis of variance (ANOVA) was conducted, followed by multiple comparisons of means using the post hoc Tukey test and Levene’s test for equal variances to evaluate the statistical significance of differences among formulations. Assumptions regarding homogeneity of variance, normality of residuals or residuals have the same distribution and independence of residuals were met. The differences were considered statistically significant at a probability less than 0.05 (*p* < 0.05). 

## 3. Results and Discussion

### 3.1. Proximate Composition of GP, SF and Pastry Products

The analytical results for the proximate composition of Merlot grape pomace powder (GP), spelt flour and pastry products are presented in Table 2. 

It can be noted that the drying of raw grape pomace at 60 °C resulted in a final moisture content of 4.872%. Drying of plant origin by-products for flour production usually takes place below 65 °C to reduce losses of phenolic compounds, anthocyanins and proteins [35,42]. 

The SF moisture was far higher than that of GP. Hence, a significant (*p* < 0.05) moisture reduction with the increasing level of GP incorporation in the pastry products was observed, reducing from 6.024 g/100 g (control) to 4.919 g/100 g (biscuits with 25% GP), 15.622 g/100 g (control) to 14.637 g/100 g (cakes with 25% GP) and from 26.163 g/100 g to 24.711 g/100 g (rolls with 25% GP). Moisture reduction in pastry products with different amounts of GP was also reported by other authors [30,31]. 

The chemical composition of GP was similar to that found by other authors who reported values of 6.4% for ash, 13.87% for protein [59] and 7.30% for lipids [60]. The data in Table 2 also show the high protein content of SF, in line with Escarnot et al. [39], who reported an average content of 14.04% at 90% dry matter content. With regard to pastry products, the changes induced in their proximate composition can be observed with a gradual increase in the level of incorporated GP from 5 to 25%.

GP-enriched formulations have higher ash and lipid content than the control, while the protein content slightly decreases. This finding is closely related to the contribution made by GP addition and has been confirmed by other authors, as follows: Troilo et al. [16] reported increases in lipid and ash content and decreases in protein when using GP in the production of functional muffins, and the same trend was revealed by Karnop et al. [31] on the physico-chemical and functional properties of cookies enriched with Bordeaux GP; Nakov et al. [30] found improvements in lipid, ash and protein content by including GP powder in cake formulations, while Acun and Gül [61] reported increased amounts of these substances in cakes when the level of GP included was 5, 10 and 15%. 

The lipid content in SF was around 3.5 times lower compared to GP, where the lipids come from the grape seeds. The increasing lipid content in GP-enriched products is a result of the abundant amount of lipids provided by the incorporated GP. Consequently, the lipid content increased from 21.788 g/100 g (control) to 22.431 g/100 g for biscuits with 25% GP, from 27.669 g/100 g (control) to 28.001 g/100 g for cakes with 25% GP and from 9.857 g/100 g (control) to 10.612 g/100 g for rolls with 25% GP incorporation. 

The content of minerals (ash) in GP was more than four times higher than in SF (6.634 vs. 1.608 g/100 g). The mineral substances provided by grape pomace led to increases in the ash content of the products: from 1.841 g/100 g (control) to a maximum of 2.290 g/100 g (biscuits with 25% GP), from 0.987 g/100 g (control) to a maximum of 1.201 g/100 g (cakes with 25% GP) and from 2.212 g/100 g (control) to a maximum of 2.761 g/100 g (rolls with 25% GP).

A slight decrease in the protein content with increasing levels of GP incorporation was observed, as GP has a lower protein content compared to SF. The improvements in protein content reported by other authors in cakes [30] are due to the fact that common wheat flour with a lower protein content than SF was used. In this situation, the incorporation of GP improved the protein content of fortified products.

The carbohydrate content of pastry formulas with different levels of GP was slightly higher compared to the control samples, with increases of 0.6–0.8 g/100 g. Small decreases in sugar content of about 0.2 g/100 g were obtained with an increasing percentage of GP, but without statistical significance (*p* > 0.05). 

The energy value was not strongly influenced by increasing the level of GP incorporation. Increases of 5.839 kcal/100 g, 4.744 kcal/100 g and 7.387 kcal/100 g were recorded in biscuits, cakes and rolls, compared to corresponding control samples, for a 25% GP inclusion level. 

The results showed the improvements in the nutritional profile of pastry products, particularly in terms of lipid and ash content, with increasing levels of grape pomace incorporation. 

### 3.2. Phytochemical Content and Antioxidant Activity of GP and SF

In the present study, the total phenolic content (TPC), total flavonoid content (TFC) and antioxidant activity evaluated by FRAP and DPPH assays were tested for SF GP, and the results are reported in Table 3. 

Phenolic compounds are the main class of bioactive substances found in SF, forming part of the plant defence system with a variety of functions [40,62]. The data in Table 3 indicate a TPC for SF of 130.211 mg GAE/100 g DW, consistent with the results of Wang et al. [40], ranging from 120.72 to 190.42 mg GAE/100 g DW, with this variability being associated with the agronomic practices, spelt wheat varieties and milling protocols. 

Our results showed that GP achieved a TPC value of 4708.683 mg GAE/100 g DW, in line with data reported by Rockenbach et al. [63], varying between 3300 and 7500 mg GAE/100 g DW. Other authors found TPC values ranging from 120 to 7480 mg GAE/100 g DW [64], 2300 to 3800 mg GAE/100 g DW [12] and 2700 to 5300 mg GAE/100 g DW [65] depending on the grape variety. Research evidence shows that the prevalent group of bioactive molecules with strong antioxidant activity in GP are phenolic compounds, mostly derived from grape skins and seeds and belonging to the class of flavonoids and nonflavonoid compounds, with a wide range of biological effects [9,10]. 

The flavonoid content of GP (3975.457 mg QE/100 g DW) was significantly higher than that of SF (81.156 mg QE/100 g DW). Similar TFC values in GP were reported by Cui et al. [66] (2833–3719 mg QE/100 g DW) and Putnik et al. [67] (3628 mg QE/100 g DW). Regarding SF, Ivanišová et al. [68] reported a TFC ranging from 33 to 155 mg QE/100 g DW, while Sumczynski et al. [69] showed a value of 37 mg QE/100 g DW.

For the antioxidant activity of GP, a value of 305.925 µM Fe^2+^/g DW by FRAP assay and 409.378 µM TE/g DW by DPPH assay was obtained. Our results for GP are well aligned with the data of Rockenbach et al. [63], ranging from 118 to 250 μmol of Fe^2+^/g DW (FRAP), respectively in the range 188–506 µM TE/g DW (DPPH). With regard to the SF, our data for FRAP (3.689 µM Fe^2+^/g DW) and DPPH (4.937 µM TE/g DW) closely match the results reported by Wang et al. [40], ranging from 2.2 to 7.4 μmol of Fe^2+^/g DW (FRAP) and from 4.5 to 13.8 µM TE/g DW (DPPH). Similar DPPH values of SF were also reported by Abdel-Aal and Rabalski [70].

The data in Table 3 reflect significantly (*p* < 0.05) higher values of TPC, TFC, FRAP and DPPH for GP compared to SF. This finding fully justifies the use of GP as a high-value substitute for SF to obtain composite flour with enhanced functionality. The production of grape pomace powder offers an attractive destination for industrial waste, as this flour has shown great potential to improve the functional value of other conventional flours or foodstuffs. As composite flours are of particular interest in the development of innovative high-quality food products, the functional properties of the component materials are a key parameter in assessing their suitability for this purpose.

### 3.3. Phytochemical Content and Antioxidant Activity of Dough and Pastry Products

In our study, the functional potential of GP was exploited in three types of pastry products, which differ both in terms of manufacturing recipe and technological process. In addition, the impact of the leavening agents (baking powder as a chemical raising agent for biscuits and cakes, and yeast as biological leavening agent for rolls) on the investigated properties is also considered. This approach integrates the raw material requirements into the assessment of the functional properties of the pastry products. 

Changes in phytochemical content and antioxidant activity following progressive supplementation of GP were tested in both dough and pastry. The phytochemical content and antioxidant activity of the dough obtained during the production process of the pastry products are shown in Table 4.

The complexity of dough composition is often overlooked when a functional material is incorporated into a bakery product, making it difficult to understand the differences that occur in the bioactive compound content of raw materials and final products. Also, the amount of functional material required to obtain a certain amount of product is often neglected when assessing the bioactive properties of finished products. The evaluation of pastry formulas in close relation to the amount of composite flour required to produce each type of product is one of the aspects that supports the added value of this approach. In our case, the amount of composite flour required for a particular pastry product has a major impact on the differences registered in the investigated properties. The data in Table 4 showed that the highest values were recorded for rolls, followed by biscuits and cakes. The values were directly correlated to the amount and composition of flour mixtures based on SF and GP required for a particular pastry formulation. 

Based on the recipe of the pastry products (Table 1), the amount of composite flour required to prepare 100 g of dough is 47.664 g for biscuits, 24.852 g for cakes and 47.902 g for rolls. The quantities of composite flours needed to prepare 100 g of products were also calculated and varied in the range of 54.828–55.473 g for biscuits, 26.754–27.067 g for cakes and 52.292–53.321 g for rolls. This justifies the lower values recorded in cake dough, considering that obtaining 100 g of product requires about 50% of the amount needed to obtain biscuits or rolls. In the case of biscuit and roll dough, if we take into account the amount of composite flour involved in the recipe, it would have been expected that close values would be obtained for the two types of dough. The higher values were basically obtained in the dough for the rolls, and this is explained by the presence of a liquid ingredient (milk) in their recipe, which led to an increase in moisture content, making the reported values per 100 g DW higher for the rolls. Within each product type, the TPC, TFC and both antioxidant tests increased significantly (*p* < 0.05) in fortified dough compared to the control with increasing levels of GP incorporation. A closer look at the three types of dough made from the same composite flour revealed significant differences (*p* < 0.05) between the functional properties. 

**Figure 3:**
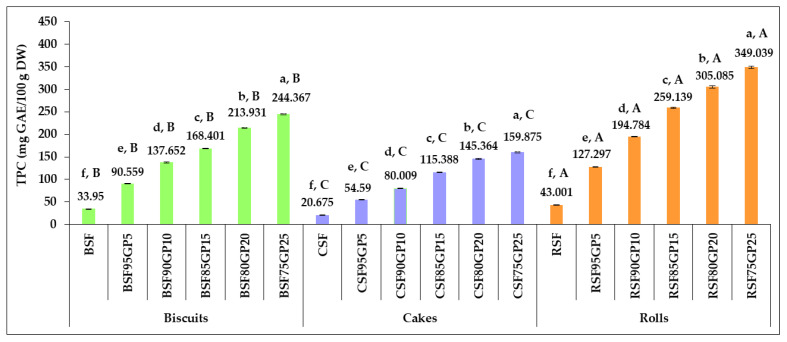
Changes in the total phenolic content of pastry formulas, in response to increasing the percentage of incorporated grape pomace. BSF, CSF, RSF (control: biscuits, cakes and rolls); BSF95GP5, CSF95GP5, RSF95GP5, (biscuits, cakes and rolls: 95% spelt flour + 5% grape pomace); BSF90GP10, CSF90GP10, RSF90GP10 (biscuits, cakes and rolls: 90% spelt flour + 10% grape pomace); BSF85GP15, CSF85GP15, RSF85GP15 (biscuits, cakes and rolls: 85% spelt flour + 15% grape pomace); BSF80GP20, CSF80GP20, RSF80GP20 (biscuits, cakes and rolls: 80% spelt flour + 20% grape pomace); BSF75GP25, CSF75GP25, RSF75GP25 (biscuits, cakes and rolls: 75% spelt flour + 25% grape pomace). The values represent the mean of three independent experiments ± standard deviation (SD). The values for bars with different letters are statistically different (one-way ANOVA, *p* < 0.05). Lowercase letters (a–f) differentiate the formulas within each pastry type, while uppercase letters (A–C) differentiate the three pastry types obtained from the same composite flour.

The TPC of the GP-enriched pastry formulas versus control samples are shown in Figure 3.

It can be seen that the increasing GP concentrations in the enriched pastry formulations led to a progressive augmentation of TPC. The highest values were noted in rolls, followed by biscuits and cakes. The TPC increased 2.667-fold, 2.640-fold and 2.960-fold as GP replacement in biscuits, cakes and rolls increased from 0% to 5%. As the level of GP incorporation increased from 0% to 25%, TPC increased 7.198-fold, 7.733-fold and 8.117-fold over the control samples. Our findings are in agreement with those of other studies that have shown increases in the TPC of pastry products when the GP level included in the recipe was increased: Nakov et al. [30] reported significant improvements in cakes as the percentage of incorporated GP powder increased from 4% to 10%, Maner et al. [71] found an improvement in the functional properties of cookies by increasing the level of added GP flour in cookies from 5% to 20%, and Karnop et al. [31] also found considerable increases in TPC by replacing the wheat flour in a cookie recipe with 20, 25 and 30% GP. Mildner-Szkudlarz et al. [26] reported increases in TPC from 211 mg GAE/100 g DM for an addition of 10% GP to 445 mg GAE/100 g DM in the case of 30% GP incorporation.

**Figure 4:**
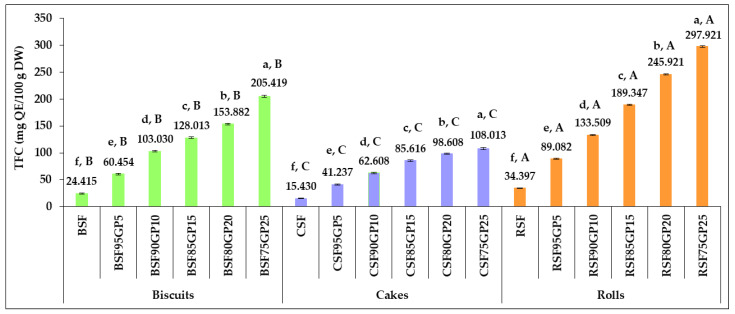
Changes in the total flavonoids content of pastry formulas, in response to increasing the percentage of incorporated grape pomace. BSF, CSF, RSF (control: biscuits, cakes and rolls); BSF95GP5, CSF95GP5, RSF95GP5, (biscuits, cakes and rolls: 95% spelt flour + 5% grape pomace); BSF90GP10, CSF90GP10, RSF90GP10 (biscuits, cakes and rolls: 90% spelt flour + 10% grape pomace); BSF85GP15, CSF85GP15, RSF85GP15 (biscuits, cakes and rolls: 85% spelt flour + 15% grape pomace); BSF80GP20, CSF80GP20, RSF80GP20 (biscuits, cakes and rolls: 80% spelt flour + 20% grape pomace); BSF75GP25, CSF75GP25, RSF75GP25 (biscuits, cakes and rolls: 75% spelt flour + 25% grape pomace). The values represent the mean of three independent experiments ± standard deviation (SD). The values for bars with different letters are statistically different (one-way ANOVA, *p* < 0.05). Lowercase letters (a–f) differentiate the formulas within each pastry type, while uppercase letters (A–C) differentiate the three pastry types obtained from the same composite flour.

The results presented in Figure 4 show that the fortification of pastry products with GP had a positive effect on the total flavonoids content, with the highest improvements achieved at a GP incorporation level of 25%.

The TFC increased 2.476-, 2.672- and 2.590-fold as GP replacement in biscuits, cakes and rolls increased from 0% to 5%. When the GP incorporation reached 25%, the TFC increased 8.414-, 7.000- and 8.661-fold in biscuits, cakes and rolls over the control samples. 

Our results are consistent with those reported by Maner et al. [71], who observed significant improvements in the TFC of cookies, in the range of 0.32–1.347 mg catechin equivalent/g, by increasing the level of grape seed powder incorporated. A similar trend was recorded in the study carried out by Nakov et al. [30], when a gradual increase in TFC was recorded by replacing wheat flour with GP at the rates of 4%, 6%, 8% and 10%. 

Significant differences (*p* < 0.05) were obtained between TPC and TFC of pastry samples with different levels of incorporation as well as between the formulations of three types of products prepared from the same composite flour.

The data in Figure 2 and Figure 3 show that an incremental increase in the level of GP incorporation in the recipe did not result in the same growth rates for TPC and TFC. Increases in the dose of GP of 2-, 3-, and 5-fold in the products did not lead to proportional increases in TPC and TFC. This finding was also reported in the studies carried out by Gaita et al. [33] and by Sęczyk et al. [72] when the changes induced in the antioxidant properties of pasta by the addition of plant origin functional materials were investigated. Several factors, including the binding of phenolic compounds to components of the food matrix, contribute to limiting the increases in their content. The incorporation of materials rich in phenolic compounds resulted in significant modifications in the relationships between the chemical constituents of farinaceous products, affecting their bioavailability. The fortification efficiency is restricted by the interactions that arise between phenolic species and the proteins in the matrix [72]. The powerful affinity of phenolic species to form different types of bonds with proteins present in the product composition can in fact result in a decline in their free forms, negatively affecting the bioavailability. The TPC is dependent on their particular ability to combine with the proteins from wheat flour or with other constituents of the matrix. On this basis, the interaction of bioactive compounds from GP with the constituents of the SF matrix should be carefully considered for the design of new pastries. 

**Figure 5:**
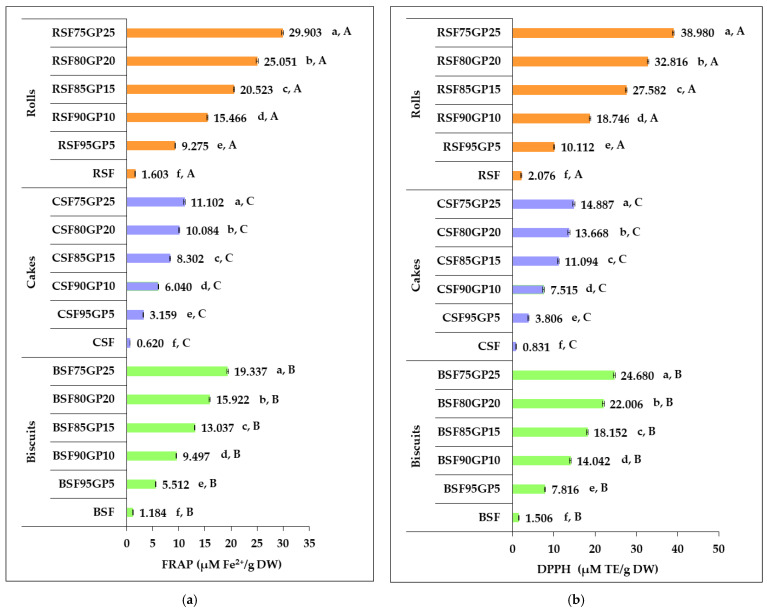
Changes in the antioxidant activity of pastry formulas in response to increasing the percentage of incorporated grape pomace: (**a**) FRAP value; (**b**) DPPH value. BSF, CSF, RSF (control: biscuits, cakes and rolls); BSF95GP5, CSF95GP5, RSF95GP5, (biscuits, cakes and rolls: 95% spelt flour + 5% grape pomace); BSF90GP10, CSF90GP10, RSF90GP10 (biscuits, cakes and rolls: 90% spelt flour + 10% grape pomace); BSF85GP15, CSF85GP15, RSF85GP15 (biscuits, cakes and rolls: 85% spelt flour + 15% grape pomace); BSF80GP20, CSF80GP20, RSF80GP20 (biscuits, cakes and rolls: 80% spelt flour + 20% grape pomace); BSF75GP25, CSF75GP25, RSF75GP25 (biscuits, cakes and rolls: 75% spelt flour + 25% grape pomace). The values represent the mean of three independent experiments ± standard deviation (SD). The values for bars with different letters are statistically different (one-way ANOVA, *p* < 0.05). Lowercase letters (a–f) differentiate the formulas within each pastry type, while uppercase letters (A–C) differentiate the three pastry types obtained from the same composite flour.

Figure 5 illustrates the changes in antioxidant properties assessed by FRAP and DPPH assays of the three types of GP-enriched pastry products compared to the control.

The antioxidant activity increased markedly by the inclusion of GP into the pastry formulation. Biscuits, cakes and rolls with different incorporation levels were found to be significantly higher (*p* < 0.05) in both FRAP and DPPH values over the control. This finding can be attributed to the increase in TPC because phenolic substances contained in winery grape waste have well-demonstrated antioxidant activities [4,5]. 

The FRAP value increases achieved in biscuits, cakes and rolls for the incorporation of 25% GP were 16.334-, 17.915- and 18.659-fold over the control samples, while the DPPH increases in BSF75GP25, CSF75GP25 and RSF75GP25 were 16.384-, 17.908- and 18.775-fold over the control samples. The samples with the highest level of GP inclusion showed the highest antioxidant activity. Similarly, Maner et al. [71] noted an increase in FRAP value with the addition of 5, 10, 15 and 20% GP in a cake recipe by 2.5, 6.4, 11 and 16 times compared to the control. Other authors [16,20,26,61] reported significant improvements in the DPPH value of bakery products with increasing GP dose. Hence, fortification with GP should allow the manufacture of pastry formulations with improved nutritional and functional properties.

With regard to the three types of products, the highest values of FRAP and DPPH were recorded in rolls, followed by biscuits and cakes, for all levels of GP incorporation. The differences recorded between the antioxidant activity of pastry formulas fortified with the same level of GP were statistically significant (*p* < 0.05).

The methods used to assess antioxidant activity differ in principle, as DPPH is used to scavenge the free radicals by phenolic compounds in GP-enriched pastries, while FRAP is used to measure the ability of antioxidant compounds in samples to reduce the ferric ions in solution to the ferrous ions, to prevent the oxidation reaction [73]. Gómez-Brandón et al. [7] stated that the phytochemicals responsible for the antioxidant activity in GP are most likely phenolic compounds, especially phenolic acids, anthocyanins and other flavonoid compounds. This finding supports the idea that increased levels of TPC and TFC in pastries, as a result of GP incorporation, strongly contribute to the enhancement of their FRAP value. The results reported by Ky et al. [74] revealed a high contribution of TPC to DPPH radical scavenging activity of grapes and grape pomaces, which indicates that the level of TPC could be a good indication of their functionality. 

All of this reinforces the idea that the ability of the formulated products to scavenge free radicals was strongly dependent on the level of TPC and TFC provided by the incorporated GP. Along with increasing TPC and TFC, a significant increase in the antioxidant activity of pastry products can be achieved. 

### 3.4. Retention Rate of Phytochemical Content and Antioxidant Activity in Pastry Products in Response to Baking

In addition to the fact that the functional attributes of pastry formulas are closely related to the amount of composite flour required for their production, of particular importance, that also supports the added value of this study, is the calculation of the retention rate of the phytochemical content and antioxidant attributes of the products, in relation to the corresponding dough, in order to assess the losses caused by the baking process. Table 5 summarises the retention rates of TPC, TFC and antioxidant activity in pastry products relative to dough as a result of baking.

TPC retention rates reported to the corresponding dough were observed in the range 41–51% for biscuits, 46–56% for cakes and 55–63% for rolls. Flavonoid retention rates were 40–51% for biscuits, 37–56% for cakes and 53–68% for rolls. The FRAP value recorded retention rates in the biscuits, cakes and rolls of 48–63%, 52–61% and 60–70%, while the DPPH value showed retention rates of 45–65%, 50–63 and 61–70%. Our results revealed significant losses of functional properties in response to heat treatment during baking, which closely match the data reported by Nakov et al. [30], revealing losses ranging from 31.19% to 49.15%. Despite the fact that baking the pastries at 180 °C induced significant losses in their functional properties, they still retained a high level of bioactive compounds. 

A significant discrepancy was observed in the retention rates of the phytochemical content and antioxidant activity of pastries made from the same composite flour in response to baking. Considering that the baking temperature for all products was 180 °C, the differences could be associated with the processes taking place in the dough. 

Dough formulations and baking conditions significantly affect the antioxidant properties and phenolic compound stability [75]. A closer look at the results in Table 5 showed a significantly higher preservation of the investigated properties in the rolls compared to the biscuits and cakes. The roll formulas leavened by yeast showed enhanced phytochemical amounts and superior antioxidant activity than the biscuit and cake formulas obtained with chemical raising agents for all levels of GP incorporation. The higher retention rates in the rolls could be assigned to the dough fermentation involved in their production. Changes in the leavening system have been found to reduce losses of phenolic compounds [76]. The leavening process is essential to develop the quality properties of pasty products. Baking powder provides a complete leavening system to produce gases via a reaction of a base like sodium bicarbonate and a weak organic acid [75]. Throughout the fermentation process developed in rolls, the gas produced by yeast activity diffuses into the dough and increases the number of air bubbles. During the first baking phase, gas bubbles are formed due to rising temperature, air expansion and carbon dioxide formation by the leavening agent [77]. Gas retention capacity is essential for dough development, significantly impacting the overall quality of the product [77]. The addition of plant-based materials rich in phenolic compounds has been shown to improve both dough functionality and gas retention by interactions between the matrix proteins and phenolic compounds [78]. According to Santetti et al. [77] and Chi and Cho [79], the addition of functional material together with the fermentation process by yeast enhances the bioavailability of phenolic compounds and the antioxidant activity of the dough. In addition, it indicates that the most effective fermentation times for the release of bioactive compounds is between 30 min and 60 min. Incorporating GP into the recipe enables an increase in dough functionality due to the biologically active compounds provided. However, more research is needed to explore the formation/degradation/hydrolysis reactions of phenolic compounds during baking.

In the case of cakes, even though the baking time was twice as long as for biscuits and rolls, this was not reflected in the impairment of their functional properties. On the one hand, this can be explained by the fact that phenolic compounds are retained by the cell matrix and can establish new types of interactions during heat treatment with other organic compounds, such as polysaccharides, which have a significant contribution to their increased stability [35,41]. On the other hand, heat treatment at elevated temperatures can potentially release the bound phenols, enhancing their availability [35]. In addition, it is known that high-temperature Maillard reaction products generated during the baking exhibit antioxidant properties [50].

The retention rate of TPC and TFC is an important criterion when considering the design of new pastry formulations with improved functional properties. It is therefore important to understand more deeply the impact of processing on these compounds in order to maximise their retention in final products. To this end, further analysis of individual polyphenolic compounds in pastry formulations with different levels of GP incorporation is needed.

### 3.5. Physical Characteristics of Pastry Products

Table 6 shows the physical characteristics of the bakery products, namely, porosity and elasticity for cakes and leavened rolls, and spread ratio for biscuits.

The results reflect decreases in elasticity and porosity of cakes and rolls with the inclusion of GP in the recipe. The maximum value for elasticity was recorded for the control cake sample (97.146%) and decreased by 5.879% for a 25% GP incorporation. In the case of the roll formulations, the decrease in elasticity was higher compared to that of the cake, being 19.870% for a 25% level of GP incorporation. A similar behaviour was recorded for porosity, which decreased by the progressive incorporation of GP. The maximum porosity value was obtained for the control sample (84.697% for cakes and 73.395% for rolls), and the values decreased compared to the control samples by 7.774% for cakes and by 9.037% in the rolls, for a 25% GP incorporation. Similar results have been reported in the literature revealing significant decreases in bread elasticity and porosity by adding non-cereal matrices at a level of 10–30%, as reported by Plustea et al. [80], who incorporated lupin flour in the bread recipe and by Dossa et al. [81], who used baobab pulp flour as a replacement for wheat flour. The structural properties of bakery products are influenced by technological parameters and the constituent phases. The elasticity and porosity are determined by dough preparation steps and, in particular, by processes involving gluten development and starch gelling. The gluten content and its ability to retain fermentation gases, as well as the amount of hydrolysable starch and its structure, influence textural parameters [80]. Considering that, by incorporating GP, the two chemical components responsible for the texture of the products are diminished, the decrease in the elasticity and porosity of enriched formulas by adding a non-cereal material is well argued.

For biscuits, the incremental incorporation of GP led to an increase in the spread ratio (SR); similar results have been reported by other authors when other non-cereal flours were added to the dough composition [1,56], probably due to the reduction in the gluten network and the increase in fibre intake [1].

### 3.6. Sensory Evaluation of Pastry Products

The sensory analysis of the pastry products was performed to assess consumer acceptability using a five-point hedonic scale, and the average scores for the attributes (appearance, aroma, texture, taste and overall acceptability) of the samples are summarised in Table 7. 

**Figure 6:**
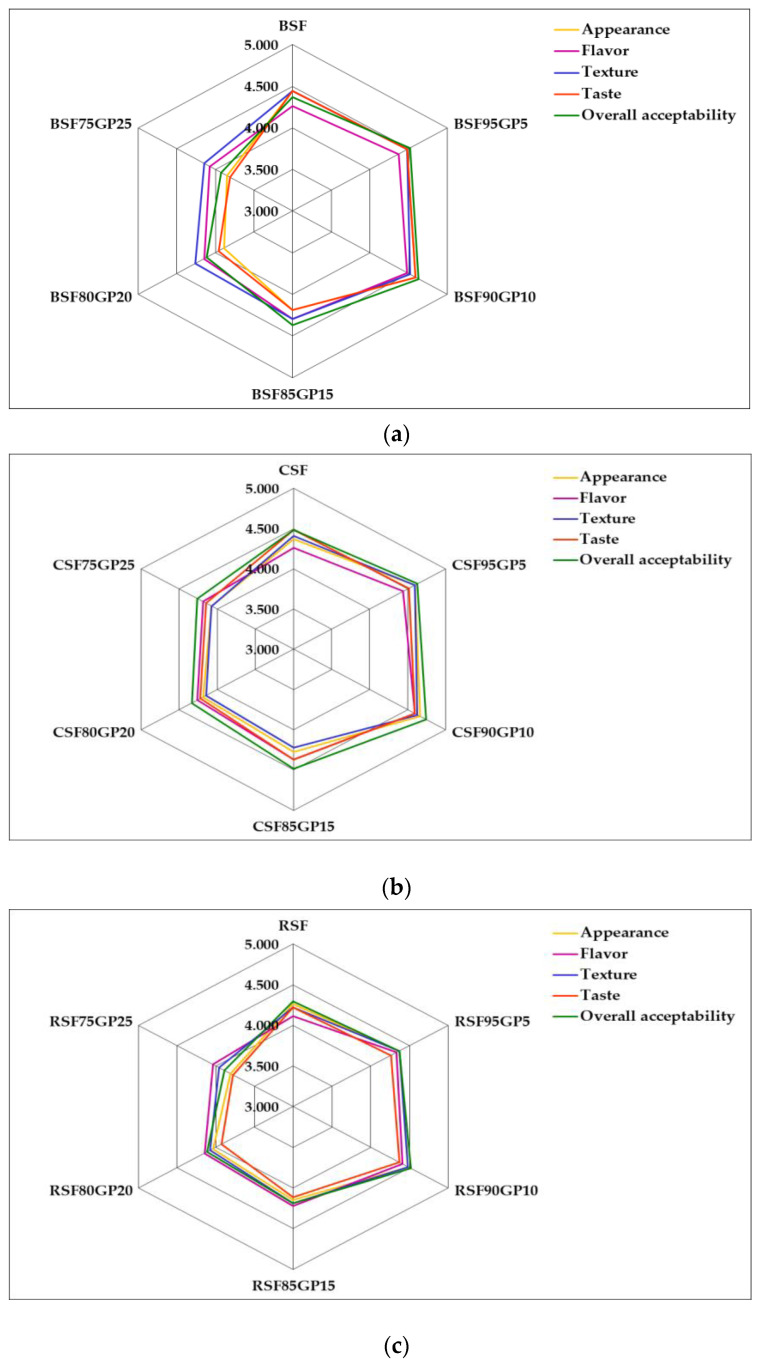
Sensory profile of quality attributes of enriched pastry formulas versus control, using a five-point hedonic scale (n = 27): (**a**): biscuits; (**b**) cakes; (**c**): rolls. BSF, CSF, RSF (control: biscuits, cakes and rolls); BSF95GP5, CSF95GP5, RSF95GP5 (biscuits, cakes and rolls: 95% spelt flour + 5% grape pomace); BSF90GP10, CSF90GP10, RSF90GP10 (biscuits, cakes and rolls: 90% spelt flour + 10% grape pomace); BSF85GP15, CSF85GP15, RSF85GP15 (biscuits, cakes and rolls: 85% spelt flour + 15% grape pomace); BSF80GP20, CSF80GP20, RSF80GP20 (biscuits, cakes and rolls: 80% spelt flour + 20% grape pomace); BSF75GP25, CSF75GP25, RSF75GP25 (biscuits, cakes and rolls: 75% spelt flour + 25% grape pomace).

The graphical representation of the sensory profile of GP-enriched pastry formulas versus control samples is illustrated in Figure 6.

In terms of the taste attribute of the biscuits, the scores increased in the following order: BSF90GP10 > BSF95GP5 > BSF > BSF85GP15 > BSF80GP20 > BSF75GP25, and in terms of flavour, the samples were ranked as follows: BSF90GP10 > BSF95GP5 > BSF85GP15 > BSF > BSF80GP20 > BSF80GP25. Regarding the overall acceptability, the score increased in the following order: BSF90GP10 > BSF95GP5 > BSF + BSF85GP15 > BSF80GP20 > BSF75GP25 (Figure 6a). The most appreciated GP-enriched biscuit formula was BSF90GP10, followed by BSF95GP5, which can be explained by the fact that the incorporation of up to 10% GP in the recipe conferred sufficient grape flavour and was appreciated by consumers. 

The results were similar to previously reported data showing that, in terms of flavour, the addition of 10% GP scored highest in the sensory analysis conducted by Lou et al. [82], also showing increased values with decreasing proportion of GP. Sharma et al. [83] highlighted that cookies with up to 15% GP recorded high sensory values, i.e., texture, mouth sensation, aroma, taste and overall acceptability. It was also pointed out that biscuits fortified with 10% red GP showed higher overall acceptability [84].

In terms of cake formulas, the most appreciated sample was CSF90GP10, followed by CSF95GP5, as for biscuits. With regard to all sensory attributes, the scores increased in the following order: CSF90GP10 > CSF95GP5 > CSF; CSF85GP15 > CSF80GP20 > CSF75GP25 (Figure 6b). The results are consistent with those reported by Nakov et al. [30], who noted that the best scores were recorded by cakes with a low level of GP addition (4%) while the samples with 6% GP showed the best texture. Incorporating GP at a level of 4–6% in cake recipes improves the nutritional value of the products and provides better sensory characteristics compared to the control sample.

In the case of GP-enriched roll formulas, the best scores for sensory attributes were also obtained for the sample with 10% GP. In terms of taste and overall acceptability, the scores of the roll samples increased in the following order: RSF90GP10 > RSF95GP5 > RSF > RSF85GP15 > RSF80GP20 > RSF75GP25 (Figure 6c). These results are in agreement with studies carried out by Boff et al. [19], who pointed out that bakery products with a maximum of 10% addition of GP flour seem to be accepted by consumers. 

Among the three types of GP-enriched pastry products, the highest scores were registered for sample CSF90GP10 with 4.667 (appearance), 4.630 (texture) and 4.630 (overall acceptability), followed by sample BSF90GP10 in terms of taste and appearance (4.593) and overall acceptability (4.630), and then roll sample RSF90GP10 with a score of 4.519 for overall acceptability, 4.485 for appearance and 4.481 for texture. 

The least appreciated samples of each product type were RSF75GP25 with a score of 3.778 for taste and 3.889 for overall acceptability, followed by BSF75GP25 with 3.815 for taste and 3.926 for overall acceptability, and CSF75GP25 with a score of 4.074 for appearance and texture and 4.185 for overall acceptability (Figure 6b). 

All pastries with GP incorporation up to 10% were rated at an extremely pleasant acceptability level (4.5–5.0). The appearance scores of all samples showed a similar trend to those reported by other authors [85], with lower scores being recorded as the level of GP incorporated increased, which could probably be explained by the darker colour of the samples. In connection with this finding, the use of GP as a natural colouring agent in bakery products has been suggested, as it has a great influence on obtaining the characteristic browned appearance of the products [7,19]. All pastry products were well accepted up to a 10% level of GP incorporation, with no significant differences between the control samples and those obtained from composite flour with 5 and 10% GP. 

## 4. Conclusions

The results of this study provide strong evidence for the use of GP as a partial replacement of spelt wheat flour to obtain fortified pastry formulas, as it is a source of phenolic compounds and has high antioxidant activity. The biscuits, cakes and rolls enriched with grape pomace powder revealed an improved nutritional profile than the control, particularly in terms of lipid and ash content. The incremental incorporation of GP up to 25% in the recipe led to significantly higher amounts of total phenolic content and total flavonoids and allowed increasing antioxidant activity compared to the control. The three types of pastry products significantly differ in their functional properties, with the highest values for rolls, followed by biscuits and cakes. This finding could be assigned to the amount of composite flour based on SF and GP included in their recipe, but also to the leavening agent involved in their production. Roll formulations prepared with yeast as a natural leavening agent revealed higher levels of TPC, TFC, FRAP and DPPH than the biscuits and cakes formulated with baking powder as a chemical raising agent. The dough fermentation process seemed to improve the functionality of pastry products and can be a determining factor for the design of new value-added formulations. The retention rate of phytochemical content and antioxidant activity of pastry products in response to baking revealed a preservation in the range of 41–63% for TPC, 37–65% for TFC, 48–70% for FRAP value and 45–70% for DPPH value relative to the corresponding dough. The porosity and elasticity of the cakes and rolls decreased via augmenting the GP level, while the spread ratio of the biscuits increased. Although GP incorporation influenced the sensory attributes of the fortified products, all pastries with a GP incorporation of up to 10% were rated at an extremely pleasant acceptability level. These data are useful as inputs in the formulation of new food products with improved functionality. The proposed use of GP, in addition to providing food with improved nutritional and functional properties, will also mitigate the environmental problems associated with their disposal.

## Figures and Tables

**Table 1:** Recipe for manufacturing biscuits, cakes and rolls (control samples and fortified formulas).

Ingredients	BSF	BSF95GP5	BSF90GP10	BSF85GP15	BSF80GP20	BSF75GP25
SF (g)	250	-	-	-	-	-
Composite flour (g)	-	250	250	250	250	250
Sugar (g)	85	85	85	85	85	85
Butter (g)	125	125	125	125	125	125
Eggs (g)	60	60	60	60	60	60
Baking powder (g)	3.500	3.500	3.500	3.500	3.500	3.500
Salt (g)	1	1	1	1	1	1
Total materials (g)	524.500	524.500	524.5	524.5	524.5	524.5
Biscuits (g)	455.967	454.724	453.718	452.821	451.833	450.668
	**CSF**	**CSF95GP5**	**CSF90GP10**	**CSF85GP15**	**CSF80GP20**	**CSF75GP25**
SF (g)	105	-	-	-	-	-
Composite flour (g)	-	105	105	105	105	105
Sugar (g)	105	105	105	105	105	105
Butter (g)	105	105	105	105	105	105
Eggs (g)	105	105	105	105	105	105
Baking powder	1.500	1.500	1.500	1.500	1.500	1.500
Salt	1	1	1	1	1	1
Total materials (g)	422.500	422.500	422.500	422.500	422.500	422.500
Cakes (g)	392.460	391.592	390.770	389.689	388.870	387.932
	**RSF**	**RSF95GP5**	**RSF90GP10**	**RSF85GP15**	**RSF80GP20**	**RSF75GP25**
SF (g)	250	-	-	-	-	-
Composite flour (g)	-	250	250	250	250	250
Sugar (g)	50	50	50	50	50	50
Milk (g)	100	100	100	100	100	100
Butter (g)	37.500	37.500	37.500	37.500	37.500	37.500
Eggs (g)	60	60	60	60	60	60
Yeast (g)	20	20	20	20	20	20
Salt (g)	4.400	4.400	4.400	4.400	4.400	4.400
Total materials (g)	521.900	523.900	521.900	521.900	521.900	521.900
Rolls (g)	478.080	476.101	474.328	472.589	470.836	468.860

SF: spelt flour; BSF, CSF, RSF (control: biscuits, cakes and rolls); BSF95GP5, CSF95GP5, RSF95GP5, (biscuits, cakes and rolls: 95% spelt flour + 5% grape pomace); BSF90GP10, CSF90GP10, RSF90GP10 (biscuits, cakes and rolls: 90% spelt flour + 10% grape pomace); BSF85GP15, CSF85GP15, RSF85GP15 (biscuits, cakes and rolls: 85% spelt flour + 15% grape pomace); BSF80GP20, CSF80GP20, RSF80GP20 (biscuits, cakes and rolls: 80% spelt flour + 20% grape pomace); BSF75GP25, CSF75GP25, RSF75GP25 (biscuits, cakes and rolls: 75% spelt flour + 25% grape pomace).

**Table 2:** Proximate composition of spelt flour, grape pomace powder and pastry products (control samples and fortified formulas).

Sample	Moisture (g/100 g)	Ash (g/100 g)	Protein (g/100 g)	Lipids (g/100 g)	Carbohydrates (g/100 g)	Sugar (g/100 g)	Energy Value (kcal/100 g)
SF	12.291 ± 0.026 ^a^	1.608 ± 0.008 ^b^	14.594 ± 0.066 ^a^	2.506 ± 0.012 ^b^	69.001	1.619 ± 0.014 ^a^	356.934
GP	4.872 ± 0.011 ^b^	6.634 ± 0.023 ^a^	13.421 ± 0.054 ^b^	8.861 ± 0.043 ^a^	66.212	1.517 ± 0.011 ^b^	398.281
Pastry products							
BSF	6.024 ± 0.030 ^a^	1.841 ± 0.006 ^f^	8.803 ± 0.027 ^a^	21.788 ± 0.071 ^e^	61.544	18.708 ± 0.075 ^a^	477.480
BSF95GP5	5.767 ± 0.034 ^b^	1.927 ± 0.010 ^e^	8.678 ± 0.039 ^b^	21.916 ± 0.066 ^e^	61.712	18.664 ± 0.066 ^a^	478.804
BSF90GP10	5.558 ± 0.037 ^c^	2.019 ± 0.014 ^d^	8.567 ± 0.033 ^c^	22.037 ± 0.068 ^d^	61.819	18.625 ± 0.053 ^a^	479.877
BSF85GP15	5.371 ± 0.028 ^d^	2.119 ± 0.011 ^c^	8.456 ± 0.040 ^d^	22.169 ± 0.075 ^c^	61.885	18.582 ± 0.070 ^a^	480.885
BSF80GP20	5.164 ± 0.022 ^e^	2.208 ± 0.012 ^b^	8.348 ± 0.032 ^e^	22.297 ± 0.056 ^b^	61.983	18.541 ± 0.061 ^a^	481.997
BSF75GP25	4.919 ± 0.025 ^f^	2.290 ± 0.015 ^a^	8.242 ± 0.038 ^f^	22.431 ± 0.080 ^a^	62.118	18.519 ± 0.073 ^a^	483.319
CSF	15.622 ± 0.039 ^a^	0.987 ± 0.003 ^f^	6.831 ± 0.034 ^a^	27.669 ± 0.057 ^c^	48.891	23.651 ± 0.089 ^a^	471.909
CSF95GP5	15.435 ± 0.054 ^b^	1.011 ± 0.005 ^e^	6.767 ± 0.033 ^a^	27.726 ± 0.071 ^c^	49.061	23.624 ± 0.069 ^a^	472.846
CSF90GP10	15.257 ± 0.037 ^c^	1.052 ± 0.007 ^d^	6.708 ± 0.025 ^b^	27.795 ± 0.054 ^c^	49.188	23.578 ± 0.075 ^a^	473.739
CSF85GP15	15.022 ± 0.046 ^d^	1.099 ± 0.011 ^c^	6.657 ± 0.037 ^c^	27.867 ± 0.079 ^c^	49.355	23.531 ± 0.065 ^a^	474.851
CSF80GP20	14.843 ± 0.050 ^e^	1.149 ± 0.009 ^b^	6.602 ± 0.027 ^d^	27.928 ± 0.084 ^b^	49.478	23.473 ± 0.073 ^a^	475.672
CSF75GP25	14.637 ± 0.036 ^f^	1.201 ± 0.012 ^a^	6.537 ± 0.034 ^e^	28.001 ± 0.098 ^a^	49.624	23.452 ± 0.083 ^a^	476.653
RSF	26.163 ± 0.046 ^a^	2.212 ± 0.006 ^f^	10.668 ± 0.040 ^a^	9.857 ± 0.025 ^f^	51.100	10.961 ± 0.076 ^a^	335.785
RSF95GP5	25.856 ± 0.050 ^b^	2.324 ± 0.005 ^e^	10.536 ± 0.046 ^b^	10.006 ± 0.030 ^e^	51.278	10.928 ± 0.063 ^a^	337.310
RSF90GP10	25.579 ± 0.059 ^c^	2.435 ± 0.009 ^d^	10.409 ± 0.034 ^b^	10.155 ± 0.026 ^d^	51.422	10.901 ± 0.058 ^a^	338.719
RSF85GP15	25.305 ± 0.064 ^d^	2.540 ± 0.010 ^c^	10.237 ± 0.058 ^c^	10.309 ± 0.041 ^c^	51.609	10.867 ± 0.055 ^a^	340.165
RSF80GP20	25.027 ± 0.046 ^e^	2.653 ± 0.008 ^b^	10.138 ± 0.051 ^c^	10.456 ± 0.038 ^b^	51.726	10.821 ± 0.076 ^a^	341.560
RSF75GP25	24.711 ± 0.053 ^f^	2.761 ± 0.011 ^a^	10.009 ± 0.047 ^d^	10.612 ± 0.027 ^a^	51.907	10.779 ± 0.081 ^a^	343.172

SF: spelt flour; GP: grape pomace; BSF, CSF, RSF (control: biscuits, cakes and rolls); BSF95GP5, CSF95GP5, RSF95GP5, (biscuits, cakes and rolls: 95% spelt flour + 5% grape pomace); BSF90GP10, CSF90GP10, RSF90GP10 (biscuits, cakes and rolls: 90% spelt flour + 10% grape pomace); BSF85GP15, CSF85GP15, RSF85GP15 (biscuits, cakes and rolls: 85% spelt flour + 15% grape pomace); BSF80GP20, CSF80GP20, RSF80GP20 (biscuits, cakes and rolls: 80% spelt flour + 20% grape pomace); BSF75GP25, CSF75GP25, RSF75GP25 (biscuits, cakes and rolls: 75% spelt flour + 25% grape pomace). The values represent the mean of three independent experiments ± standard deviation (SD). The values with different superscripts in a column are statistically different (one-way ANOVA, *p* < 0.05).

**Table 3:** Total phenolic content, total flavonoids content and antioxidant activity of spelt flour and grape pomace powder.

Sample	TPC (mg GAE/100 g DW)	TFC (mg QE/100 g DW)	FRAP (µM Fe^2+^/g DW)	DPPH (µM TE/g DW)
SF	130.211 ± 0.584 ^b^	81.156 ± 0.437 ^b^	3.689 ± 0.028 ^b^	4.937 ± 0.041 ^b^
GP	4708.683 ± 4.053 ^a^	3975.457 ± 3.291 ^a^	305.925 ± 1.817 ^a^	409.378 ± 2.019 ^a^

SF: spelt flour; GP: grape pomace powder. The values represent the mean of three independent experiments ± standard deviation (SD). The values with different superscripts in a column are statistically different (one-way ANOVA, *p* < 0.05).

**Table 4:** Total phenol content, total flavonoid content and antioxidant activity of dough (control samples and fortified formulas).

Sample	TPC (mg GAE/100 g DW)	TFC (mg QE/100 g DW)	FRAP (µM Fe^2+^/g DW)	DPPH (µM TE/g DW)
DBSF	66.323 ± 0.362 ^f, B^	48.137 ± 0.294 ^f, B^	1.879 ± 0.024 ^f, B^	2.515 ± 0.029 ^f, B^
DBSF95GP5	180.267± 0.767 ^e, B^	132.465 ± 0.604 ^e, B^	8.917 ± 0.108 ^e, B^	11.978 ± 0.143 ^e, B^
DBSF90GP10	291.244 ± 1.486 ^d, B^	216.605 ± 1.211 ^d, B^	16.138 ± 0.173 ^d, B^	22.518 ± 0.181 ^d, B^
DBSF85GP15	397.101 ± 1.877 ^c, B^	302.448 ± 1.547 ^c, B^	24.109 ± 0.208 ^c, B^	32.772 ± 0.193 ^c, B^
DBSF80GP20	500.551 ± 1.935 ^b, B^	384.141 ± 1.628 ^b, B^	32.259 ± 0.246 ^b, B^	42.841 ± 0.260 ^b, B^
DBSF75GP25	599.799 ± 2.104 ^a, B^	479.135 ± 1.714 ^a, B^	40.200 ± 0.361 ^a, B^	54.256 ± 0.412 ^a, B^
DCSF	37.871 ± 0.275 ^f, C^	27.501 ± 0.224 ^f, C^	1.017 ± 0.021 ^f, C^	1.361 ± 0.025 ^f, C^
DCSF95GP5	98.084 ± 0.439 ^e, C^	73.841 ± 0.331 ^e, C^	5.401 ± 0.059 ^e, C^	6.481 ± 0.073 ^e, C^
DCSF90GP10	158.134 ± 0.501 ^d, C^	127.487 ± 0.593 ^d, C^	9.887 ± 0.115 ^d, C^	12.185 ± 0.136 ^d, C^
DCSF85GP15	222.448 ± 0.547 ^c, C^	181.512 ± 0.602 ^c, C^	13.864 ± 0.137 ^c, C^	17.733 ± 0.159 ^c, C^
DCSF80GP20	282.755 ± 1.219 ^b, C^	238.541 ± 0.903 ^b, C^	17.586 ± 0.154 ^b, C^	23.181 ± 0.178 ^b, C^
DCSF75GP25	348.479 ± 1.425 ^a, C^	290.651 ± 1.071 ^a, C^	21.265 ± 0.188 ^a, C^	29.358 ± 0.185 ^a, C^
DRSF	73.128 ± 0.463 ^f, A^	53.128 ± 0.342 ^f, A^	2.279 ± 0.031 ^f, A^	3.049 ± 0.037 ^f, A^
DRSF95GP5	201.818 ± 0.854 ^e, A^	157.487 ± 0.611 ^e, A^	13.223 ± 0.147 ^e, A^	14.526 ± 0.147 ^e, A^
DRSF90GP10	314.397 ± 1.207 ^d, A^	251.012 ± 1.004 ^d, A^	22.157 ± 0.164 ^d, A^	27.307 ± 0.191 ^d, A^
DRSF85GP15	426.532 ± 1.446 ^c, A^	358.009 ± 1.317 ^c, A^	31.070 ± 0.183 ^c, A^	39.742 ± 0.239 ^c, A^
DRS0GP20	535.685 ± 1.913 ^b, A^	466.108 ± 1.629 ^b, A^	40.961 ± 0.209 ^b, A^	51.952 ± 0.308 ^b, A^
DRSF75GP25	630.041 ± 2.307 ^a, A^	562.083 ± 1.853 ^a, A^	49.6674 ± 0.252 ^a, A^	63.795 ± 0.363 ^a, A^

DBSF, DCSF, DRSF (control dough: biscuits, cakes and rolls); DBSF95GP5, DCSF95GP5, DRSF95GP5, (dough for biscuits, cakes and rolls: 95% spelt flour + 5% grape pomace); DBSF90GP10, DCSF90GP10, RRSF90GP10 (dough for biscuits, cakes and rolls: 90% spelt flour + 10% grape pomace); DBSF85GP15, DCSF85GP15, DRSF85GP15 (dough for biscuits, cakes and rolls: 85% spelt flour + 15% grape pomace); DBSF80GP20, DCSF80GP20, DRSF80GP20 (dough for biscuits, cakes and rolls: 80% spelt flour + 20% grape pomace); DBSF75GP25, DCSF75GP25, DRSF75GP25 (dough for biscuits, cakes and rolls: 75% spelt flour + 25% grape pomace). The values represent the mean of three independent experiments ± standard deviation (SD). Values with different superscripts in a column are statistically different (one-way ANOVA, *p* < 0.05). Lowercase letters (a–f) differentiate the formulas within each dough type, while uppercase letters (A–C) differentiate the three types of dough obtained from the same composite flour.

**Table 5:** Retention rate of phytochemical content and antioxidant activity of pastry products in response to baking.

Sample	Retention Rate of TPC (%)	Retention Rate of TFC (%)	Retention Rate of FRAP (%)	Retention Rate of DPPH (%)
BSF	51.189 ± 0.125 ^a, C^	50.721 ± 0.114 ^a, C^	63.006 ± 0.154 ^a, B^	59.905 ± 0.147 ^c, C^
BSF95GP5	50.236 ± 0.153 ^b, C^	45.638 ± 0.138 ^c, C^	61.811 ± 0.189 ^b, B^	65.254 ± 0.161 ^a, B^
BSF90GP10	47.263 ± 0.102 ^c, C^	47.566 ± 0.103 ^b, C^	58.849 ± 0.127 ^c, C^	62.360 ± 0.134 ^b, B^
BSF85GP15	42.408 ± 0.129 ^d, C^	42.326 ± 0.129 ^d, C^	54.075 ± 0.165 ^d, C^	55.389 ± 0.169 ^d, C^
BSF80GP20	42.739 ± 0.121 ^d, C^	40.059 ± 0.113 ^e, C^	49.356 ± 0.140 ^e, C^	51.367 ± 0.146 ^e, C^
BSF75GP25	40.741 ± 0.097 ^e, C^	42.873 ± 0.102 ^f, B^	48.102 ± 0.111 ^f, C^	45.488 ± 0.107 ^f, C^
CSF	54.593 ± 0.132 ^b, B^	56.108 ± 0.137 ^a, B^	60.954 ± 0.149 ^a, C^	61.099 ±0.151 ^c, B^
CSF95GP5	55.656 ± 0.170 ^a, B^	55.846 ± 0.158 ^a, B^	58.489 ± 0.178 ^c, C^	58.714 ±0.179 ^d, C^
CSF90GP10	50.596 ± 0.117 ^e, B^	49.110 ± 0.108 ^b, B^	61.090 ± 0.132 ^a, B^	61.677 ±0.133 ^b, C^
CSF85GP15	51.872 ± 0.158 ^c, B^	47.168 ± 0.144 ^c, B^	59.882 ± 0.183 ^b, B^	62.558 ±0.172 ^a, B^
CSF80GP20	51.410 ± 0.146 ^d, B^	41.338 ± 0.118 ^d, B^	57.334 ± 0.162 ^d, B^	58.960 ±0.167 ^d, B^
CSF75GP25	45.878 ± 0.109 ^f, B^	37.163 ± 0.107 ^e, C^	52.209 ± 0.143 ^e, B^	50.709 ±0.124 ^e, B^
RSF	58.802 ± 0.144 ^d, A^	64.744 ± 0.159 ^a, A^	70.332 ± 0.172 ^a, A^	68.087 ± 0.167 ^c, A^
RSF95GP5	63.075 ± 0.172 ^a, A^	56.564 ± 0.173 ^b, A^	70.144 ± 0.184 ^a, A^	69.617 ± 0.149 ^a, A^
RSF90GP10	61.955 ± 0.134 ^b, A^	53.188 ± 0.137 ^c, A^	69.801 ± 0.150 ^b, A^	68.647 ± 0.152 ^b, A^
RSF85GP15	60.755 ± 0.179 ^c, A^	52.889 ± 0.143 ^c, A^	66.052 ± 0.167 ^c, A^	69.403 ± 0.132 ^a, A^
RS0GP20	56.952 ± 0.161 ^e, A^	52.761 ± 0.160 ^c, A^	61.157 ± 0.173 ^d, A^	63.166 ± 0.154 ^d, A^
RSF75GP25	55.399 ± 0.133 ^f, A^	53.003 ± 0.164 ^c, A^	60.198 ± 0.143 ^e, A^	61.102 ± 0.140 ^e, A^

BSF, CSF, RSF (control: biscuits, cakes and rolls); BSF95GP5, CSF95GP5, RSF95GP5, (biscuits, cakes and rolls: 95% spelt flour + 5% grape pomace powder); BSF90GP10, CSF90GP10, RSF90GP10 (biscuits, cakes and rolls: 90% spelt flour + 10% grape pomace); BSF85GP15, CSF85GP15, RSF85GP15 (biscuits, cakes and rolls: 85% spelt flour + 15% grape pomace); BSF80GP20, CSF80GP20, RSF80GP20 (biscuits, cakes and rolls: 80% spelt flour + 20% grape pomace); BSF75GP25, CSF75GP25, RSF75GP25 (biscuits, cakes and rolls: 75% spelt flour + 25% grape pomace). The values represent the mean of three independent experiments ± standard deviation (SD). Data with different superscripts in a column are statistically different (one-way ANOVA, *p* < 0.05). Lowercase letters (a–f) differentiate the formulas within each pastry type, while uppercase letters (A–C) differentiate the three pastry types obtained from the same composite flour.

**Table 6:** Physical characteristics of pastry products (control samples and fortified formulas).

Physical Characteristics	Pastry Products					
	BSF	BSF95GP5	BSF90GP10	BSF85GP15	BSF80GP20	BSF75GP25
SR	5.001 ± 0.012 ^e^	5.109 ± 0.011 ^d^	5.158 ± 0.013 ^c^	5.206 ± 0.010 ^b^	5.229 ± 0.013 ^a^	5.257 ± 0.011 ^a^
	CSF	CSF95GP5	CSF90GP10	CSF85GP15	CSF80GP20	CSF75GP25
Porosity (%)	84.697 ± 0.282 ^a^	84.431 ± 0.273 ^a^	83.224 ± 0.258 ^b^	82.877 ± 0.249 ^b^	80.149 ± 0.227 ^c^	78.113 ± 0.221 ^d^
Elasticity (%)	97.146 ± 0.301 ^a^	97.148 ± 0.294 ^a^	95.653 ± 0.285 ^b^	94.129 ± 0.279 ^c^	92.181 ± 0.232 ^d^	91.435 ± 0.229 ^e^
	RSF	RSF95GP5	RSF90GP10	RSF85GP15	RS0GP20	RSF75GP25
Porosity (%)	73.395 ± 0.223 ^a^	68.403 ± 0.207 ^b^	68.226 ± 0.198 ^b^	67.724 ± 0.186 ^c^	67.431 ± 0.173 ^c^	66.762 ± 0.169 ^d^
Elasticity (%)	75.008 ± 0.249 ^a^	72.229 ± 0.233 ^b^	68.903 ± 0.219 ^c^	62.511 ± 0.188 ^d^	61.507 ± 0.164 ^e^	60.104 ± 0.151 ^f^

SR: spread ratio; BSF, CSF, RSF (control: biscuits, cakes and rolls); BSF95GP5, CSF95GP5, RSF95GP5, (biscuits, cakes and rolls: 95% spelt flour + 5% grape pomace powder); BSF90GP10, CSF90GP10, RSF90GP10 (biscuits, cakes and rolls: 90% spelt flour + 10% grape pomace); BSF85GP15, CSF85GP15, RSF85GP15 (biscuits, cakes and rolls: 85% spelt flour + 15% grape pomace); BSF80GP20, CSF80GP20, RSF80GP20 (biscuits, cakes and rolls: 80% spelt flour + 20% grape pomace); BSF75GP25, CSF75GP25, RSF75GP25 (biscuits, cakes and rolls: 75% spelt flour + 25% grape pomace). The values represent the mean of three independent experiments ± standard deviation (SD). Data with different superscripts in a row are statistically different (one-way ANOVA, *p* < 0.05).

**Table 7:** Global values of the sensory attributes of enriched pastry formulas versus control using a five-point hedonic scale.

Sample	Scores (5-Point Hedonic Scale)				
	Appearance	Flavour	Texture	Taste	Overall Acceptability
BSF	4.444 ± 0.506 ^a^	4.259 ± 0.447 ^a^	4.444 ± 0.506 ^a^	4.444 ± 0.506 ^a^	4.370 ± 0.565 ^a^
BSF95GP5	4.481 ± 0.509 ^a^	4.370 ± 0.492 ^a^	4.481 ± 0.509 ^a^	4.481 ± 0.509 ^a^	4.519 ± 0.509 ^a^
BSF90GP10	4.593 ± 0.501 ^a^	4.481 ± 0.509 ^a^	4.519 ± 0.509 ^a^	4.593 ± 0.501 ^a^	4.630 ± 0.492 ^a^
BSF85GP15	4.185 ± 0.396 ^b^	4.296 ± 0.465 ^a^	4.296 ± 0.465 ^a^	4.185 ± 0.483 ^b^	4.370 ± 0.492 ^a^
BSF80GP20	3.889 ± 0.320 ^c^	4.148 ± 0.362 ^a^	4.259 ± 0.447 ^a^	3.963 ± 0.338 ^c^	4.111 ± 0.320 ^b^
BSF75GP25	3.852 ± 0.362 ^c^	4.074 ± 0.267 ^b^	4.148 ± 0.362 ^b^	3.815 ± 0.396 ^d^	3.926 ± 0.267 ^c^
CSF	4.370 ± 0.492 ^a^	4.259 ± 0.447 ^a^	4.407 ± 0.501 ^a^	4.481 ± 0.509 ^a^	4.481 ± 0.509 ^a^
CSF95GP5	4.593 ± 0.501 ^a^	4.444 ± 0.506 ^a^	4.593 ± 0.501 ^a^	4.519 ± 0.509 ^a^	4.556 ± 0.506 ^a^
CSF90GP10	4.667 ± 0.480 ^a^	4.593 ± 0.501 ^a^	4.630 ± 0.492 ^a^	4.593 ± 0.501 ^a^	4.630 ± 0.492 ^a^
CSF85GP15	4.296 ± 0.465 ^b^	4.370 ± 0.492 ^a^	4.222 ± 0.424 ^b^	4.370 ± 0.492 ^a^	4.481 ± 0.509 ^a^
CSF80GP20	4.185 ± 0.483 ^c^	4.259 ± 0.526 ^a^	4.148 ± 0.362 ^b^	4.222 ± 0.424 ^b^	4.259 ± 4.259 ^a^
CSF75GP25	4.074 ± 0.267 ^c^	4.185 ± 0.396 ^b^	4.074 ± 0.385 ^b^	4.148 ± 0.362 ^c^	4.185 ± 0.396 ^b^
RSF	4.259 ± 0.447 ^a^	4.111 ± 0.320 ^a^	4.222 ± 0.424 ^a^	4.222 ± 0.424 ^a^	4.296 ± 0.465 ^a^
RSF95GP5	4.370 ± 0.492 ^a^	4.333 ± 0.480 ^a^	4.370 ± 0.492 ^a^	4.259 ± 0.447 ^a^	4.370 ± 0.492 ^a^
RSF90GP10	4.485 ± 0.509 ^a^	4.407 ± 0.501 ^a^	4.481 ± 0.509 ^a^	4.370 ± 0.492 ^a^	4.519 ± 0.509 ^a^
RSF85GP15	4.148 ± 0.362 ^b^	4.222 ± 0.424 ^a^	4.185 ± 0.396 ^a^	4.111 ± 0.320 ^a^	4.185 ± 0.396 ^b^
RS0GP20	4.037 ± 0.192 ^b^	4.148 ± 0.362 ^a^	4.074 ± 0.267 ^b^	3.926 ± 0.267 ^b^	4.111 ± 0.320 ^b^
RSF75GP25	3.815 ± 0.396 ^c^	4.037 ± 0.192 ^b^	3.963 ± 0.338 ^c^	3.778 ± 0.424 ^c^	3.889 ± 0.320 ^c^

BSF, CSF, RSF (control: biscuits, cakes and rolls); BSF95GP5, CSF95GP5, RSF95GP5, (biscuits, cakes and rolls: 95% spelt flour + 5% grape pomace); BSF90GP10, CSF90GP10, RSF90GP10 (biscuits, cakes and rolls: 90% spelt flour + 10% grape pomace); BSF85GP15, CSF85GP15, RSF85GP15 (biscuits, cakes and rolls: 85% spelt flour + 15% grape pomace); BSF80GP20, CSF80GP20, RSF80GP20 (biscuits, cakes and rolls: 80% spelt flour + 20% grape pomace); BSF75GP25, CSF75GP25, RSF75GP25 (biscuits, cakes and rolls: 75% spelt flour + 25% grape pomace). The values represent the mean of three independent experiments ± standard deviation (SD). The different letters (a–d) shown in the same column for each sensory attribute represent statistically significant differences among pastry formulas (one-way ANOVA, *p* < 0.05).

## Data Availability

The report of the analyses performed for the samples in the paper can be found at the Interdisciplinary Research Platform (PCI) at the University of Life Sciences “King Michael I”, Timisoara.

## References

[1] Nakov G., Brandolini A., Estivi L., Bertuglia K., Ivanova N., Jukić M., Komlenić D.K., Lukinac J., Hidalgo A. (2022). Effect of Tomato Pomace Addition on Chemical, Technological, Nutritional, and Sensorial Properties of Cream Crackers. Antioxidants.

[2] Trigo J.P., Alexandre E.M., Saraiva J.A., Pintado M.E. (2022). High value-added compounds from fruit and vegetable by-products–Characterization, bioactivities, and application in the development of novel food products. Crit. Rev. Food Sci. Nutr..

[3] Antonic B., Dordevic D., Jancikova S., Holeckova D., Tremlova B., Kulawik P. (2021). Effect of Grape Seed Flour on the Antioxidant Profile, Textural and Sensory Properties of Waffles. Processes.

[4] Tournour H.H., Segundo M.A., Magalhaes L.M., Barreiros L., Queiroz J., Cunha L.M. (2015). Valorization of grape pomace: Extraction of bioactive phenolics with antioxidant properties. Ind. Crops Prod..

[5] Lau K.Q., Sabran M.R., Shafie S.R. (2021). Utilization of vegetable and fruit by-products as functional ingredient and food. Front. Nutr..

[6] Beres C., Costa G.N., Cabezudo I., Da Silva-James N.K., Teles A.S., Cruz A.P., Mellinger-Silva C., Tonon R.V., Cabral L.M., Freitas S.P. (2017). Towards integral utilization of grape pomace from winemaking process: A review. Waste Manag..

[7] Gómez-Brandón M., Lores M., Insam H., Domínguez J. (2019). Strategies for recycling and valorization of grape marc. Crit. Rev. Biotechnol..

[8] Iuga M., Mironeasa S. (2020). Potential of grape byproducts as functional ingredients in baked goods and pasta. Compr. Rev. Food Sci. Food Saf..

[9] Yu J., Ahmedna M. (2013). Functional components of grape pomace: Their composition, biological properties and potential applications. Int. J. Food Sci. Technol..

[10] García-Lomillo J., González-SanJosé M.L. (2017). Applications of wine pomace in the food industry: Approaches and functions. Compr. Rev. Food Sci. Food Saf..

[11] Pinelo M., Rubilar M., Jerez M., Sineiro J., Núñez M.J. (2005). Effect of solvent, temperature, and solvent-to-solid ratio on the total phenolic content and antiradical activity of extracts from different components of grape pomace. J. Agric. Food Chem..

[12] Monteiro G.C., Minatel I.O., Junior A.P., Gomez-Gomez H.A., de Camargo J.P.C., Diamante M.S., Pereira Basilio L.S., Tecchio M.A., Lima G.P.P. (2021). Bioactive compounds and antioxidant capacity of grape pomace flours. LWT-Food Sci. Technol..

[13] Ferrer-Gallego R., Silva P. (2022). The Wine Industry By-Products: Applications for Food Industry and Health Benefits. Antioxidants.

[14] Bender A.B., Speroni C.S., Salvador P.R., Loureiro B.B., Lovatto N.M., Goulart F.R., Lovattoc M.T., Mirandad M.Z., Silvab L.P., Penna N.G. (2017). Grape pomace skins and the effects of its inclusion in the technological properties of muffins. J. Culin. Sci. Technol..

[15] Chowdhary P., Gupta A., Gnansounou E., Pandey A., Chaturvedi P. (2021). Current trends and possibilities for exploitation of Grape pomace as a potential source for value addition. Environ. Pollut..

[16] Troilo M., Difonzo G., Paradiso V.M., Pasqualone A., Caponio F. (2022). Grape Pomace as Innovative Flour for the Formulation of Functional Muffins: How Particle Size Affects the Nutritional, Textural and Sensory Properties. Foods.

[17] Muhlack R.A., Potumarthi R., Jeffery D.W. (2018). Sustainable wineries through waste valorisation: A review of grape marc utilisation for value-added products. Waste Manag..

[18] Torbica A., Škrobot D., Hajnal E.J., Belović M., Zhang N. (2019). Sensory and physico-chemical properties of wholegrain wheat bread prepared with selected food by-products. LWT-Food Sci. Technol..

[19] Boff J.M., Strasburg V.J., Ferrari G.T., de Oliveira Schmidt H., Manfroi V., de Oliveira V.R. (2022). Chemical, Technological, and Sensory Quality of Pasta and Bakery Products Made with the Addition of Grape Pomace Flour. Foods.

[20] Hayta M., Özuğur G., Etgü H., Şeker İ.T. (2014). Effect of Grape (*Vitis vinifera* L.) Pomace on the Quality, Total Phenolic Content and Anti-Radical Activity of Bread. J. Food Process. Preserv..

[21] Šporin M., Avbelj M., Kovač B., Možina S.S. (2018). Quality characteristics of wheat flour dough and bread containing grape pomace flour. Food Sci. Technol. Int..

[22] Rosales Soto M.U., Brown K., Ross C.F. (2012). Antioxidant activity and consumer acceptance of grape seed flour-containing food products. Int. J. Food Sci. Technol..

[23] Samohvalova O., Grevtseva N., Brykova T., Grigorenko A. (2016). The effect of grape seed powder on the quality of butter biscuits. East. Eur. J. Enterpr. Technol..

[24] Meral R., Doğan İ.S. (2013). Grape seed as a functional food ingredient in bread-making. Int. J. Food Sci. Nutr..

[25] Tolve R., Simonato B., Rainero G., Bianchi F., Rizzi C., Cervini M., Giuberti G. (2021). Wheat Bread Fortification by Grape Pomace Powder: Nutritional, Technological, Antioxidant, and Sensory Properties. Foods.

[26] Mildner-Szkudlarz S., Bajerska J., Zawirska-Wojtasiak R., Górecka D. (2013). White grape pomace as a source of dietary fibre and polyphenols and its effect on physical and nutraceutical characteristics of wheat biscuits. J. Sci. Food Agric..

[27] Palma M.L., Nunes M.C., Gameiro R., Rodrigues M., Gothe S., Tavares N., Pego C., Nicolai M., Pereira P. (2020). Preliminary sensory evaluation of salty crackers with grape pomace flour. Biomed. Biopharm. Res..

[28] Sant’Anna V., Christiano F.D.P., Marczak L.D.F., Tessaro I.C., Thys R.C.S. (2014). The effect of the incorporation of grape marc powder in fettuccini pasta properties. LWT-Food Sci. Technol..

[29] Oliveira B.E., Contini L., Garcia V.A.D.S., Cilli L.P.D.L., Chagas E.G.L., Andreo M.A., Vanin F.M., Carvalho R.A., Sinnecker P., Venturini A.C. (2022). Valorization of grape by-products as functional and nutritional ingredients for healthy pasta development. J. Food Process. Preserv..

[30] Nakov G., Brandolini A., Hidalgo A., Ivanova N., Stamatovska V., Dimov I. (2020). Effect of grape pomace powder addition on chemical, nutritional and technological properties of cakes. LWT-Food Sci. Technol..

[31] Karnopp A.R., Figueroa A.M., Los P.R., Teles J.C., Simões D.R.S., Barana A.C., Kubiaki F.T., Oliveira J.G.B.d., Granato D. (2015). Effects of whole-wheat flour and bordeaux grape pomace (*Vitis labrusca* L.) on the sensory, physicochemical and functional properties of cookies. Food Sci. Technol..

[32] Fontana M., Murowaniecki Otero D., Pereira A.M., Santos R.B., Gularte M.A. (2022). Grape Pomace Flour for Incorporation into Cookies: Evaluation of Nutritional, Sensory and Technological Characteristics. J. Culin. Sci. Technol..

[33] Gaita C., Alexa E., Moigradean D., Conforti F., Poiana M.A. (2020). Designing of high value-added pasta formulas by incorporation of grape pomace skins. Rom. Biotechnol. Lett..

[34] Rainero G., Bianchi F., Rizzi C., Cervini M., Giuberti G., Simonato B. (2022). Breadstick fortification with red grape pomace: Effect on nutritional, technological and sensory properties. J. Sci. Food Agric..

[35] Larrosa A.P.Q., Otero D.M. (2021). Flour made from fruit by-products: Characteristics, processing conditions, and applications. J. Food Process. Preserv..

[36] Hasmadi M., Noorfarahzilah M., Noraidah H., Zainol M.K., Jahurul M.H.A. (2020). Functional properties of composite flour: A review. Food Res..

[37] Kohajdová Z., Karovicova J. (2008). Nutritional value and baking application of spelt wheat. Acta Sci. Pol. Technol. Aliment..

[38] Biel W., Stankowski S., Jaroszewska A., Pużyński S., Bośko P. (2016). The influence of selected agronomic factors on the chemical composition of spelt wheat (*Triticum aestivum* ssp. *spelta* L.) grain. J. Integr. Agric..

[39] Escarnot E., Jacquemin J.M., Agneessens R., Paquot M. (2012). Comparative study of the content and profiles of macronutrients in spelt and wheat, a review. Biotechnol. Agron. Soc. Environ..

[40] Wang J., Chatzidimitriou E., Wood L., Hasanalieva G., Markellou E., Iversen P.O., Seala C., Baranskib M., Vigarj V., Ernstj L. (2020). Effect of wheat species (*Triticum aestivum* vs. *T. spelta*), farming system (organic vs. conventional) and flour type (wholegrain vs white) on composition of wheat flour–Results of a retail survey in the UK and Germany–2. Antioxidant activity, and phenolic and mineral content. Food Chem..

[41] Jung J., Cavender G., Zhao Y. (2015). Impingement drying for preparing dried apple pomace flour and its fortification in bakery and meat products. J. Food Sci. Technol..

[42] Santos D., da Silva J.A.L., Pintado M. (2022). Fruit and vegetable by-products’ flours as ingredients: A review on production process, health benefits and technological functionalities. LWT-Food Sci. Technol..

[43] (2003). Microbiology of Food and Animal Feeding Stuffs—Horizontal Method for the Enumeration of Microorganisms—Colony-Count Technique at 30 Degrees C.

[44] (2004). Microbiology of Food and Animal Feeding Stuffs—Horizontal Methods for the Detection and Enumeration of Enterobacteriaceae—Part 2: Colony-Count Method.

[45] (2008). Microbiology of Food and Animal Feeding Stuffs—Horizontal Method for the Enumeration of Yeasts and Moulds—Part 2: Colony Count Technique in Products with Water Activity Less than or Equal to 0.95.

[46] (2006). Microbiology of Food and Animal Feeding Stuffs—Horizontal Method for the Determination of Low Numbers of Presumptive Bacillus cereus—Most Probable Number Technique and Detection Method.

[47] Commission Regulation (EC) No 2073/2005 of 15 November 2005 on Microbiological Criteria for Foodstuffs. https://www.eumonitor.eu/9353000/1/j4nvk6yhcbpeywk_j9vvik7m1c3gyxp/vi8rm2zgvzuf.

[48] Association of Official Analytical Chemists (AOAC) (2000). Official Methods of Analysis.

[49] Das P.C., Khan M.J., Rahman M.S., Majumder S., Islam M.N. (2019). Comparison of the physico-chemical and functional properties of mango kernel flour with wheat flour and development of mango kernel flour based composite cakes. NFS J..

[50] Litwinek D., Gumul D., Łukasiewicz M., Zięba T., Kowalski S. (2023). The Effect of Red Potato Pulp Preparation and Stage of Its Incorporation into Sourdough or Dough on the Quality and Health-Promoting Value of Bread. Appl. Sci..

[51] Blanch G.P., Ruiz del Castillo M.L. (2021). Effect of Baking Temperature on the Phenolic Content and Antioxidant Activity of Black Corn (*Zea mays* L.) Bread. Foods.

[52] Al-Farsi M., Al-Amri A., Al-Hadhrami A., Al-Belushi S. (2018). Color, flavonoids, phenolics and antioxidants of Omani honey. Heliyon.

[53] Mekky H., El Sohafy S., Abu El-Khair R.A., El Hawiet A.E. (2017). Total polyphenolic content and antioxidant activity of Silybum marianum cultures grown on different growth regulators. Int. J. Pharm. Pharm. Sci..

[54] Metzner Ungureanu C.-R., Poiana M.-A., Cocan I., Lupitu A.I., Alexa E., Moigradean D. (2020). Strategies to Improve the Thermo-Oxidative Stability of Sunflower Oil by Exploiting the Antioxidant Potential of Blueberries Processing Byproducts. Molecules.

[55] (2007). Romanian Standard for Bread, Confectionery and Bakery Specialties—Methods of Analysis.

[56] Raymundo A., Fradinho P., Nunes M.C. (2014). Effect of Psyllium fibre content on the textural and rheological characteristics of biscuit and biscuit dough. Bioact. Carbohydr. Diet. Fibre.

[57] Alfonsi A., Coles D., Hasle C., Koppel J., Ladikas M., Schmucker von Koch J., Schroeder D., Sprumont D., Verbeke W., Zaruk D. (2012). Guidance Note: Ethics and Food-Related Research.

[58] Pestorić M., Škrobot D., Žigon U., Šimurina O., Filipčev B., Belović M., Mišan A. (2017). Sensory profile and preference mapping of cookies enriched with medicinal herbs. Int. J. Food Prop..

[59] Beres C., Freitas S.P., de Oliveira Godoy R.L., de Oliveira D.C.R., Deliza R., Iacomini M., Mellinger-Silva C., Cabral L.M.C. (2019). Antioxidant dietary fibre from grape pomace flour or extract: Does it make any difference on the nutritional and functional value?. J. Funct. Foods.

[60] Yi C., Shi J., Kramer J., Xue S., Jiang Y., Zhang M., Ma I., Pohorly J. (2009). Fatty acid composition and phenolic antioxidants of winemaking pomace powder. Food Chem..

[61] Acun S., Gül H. (2014). Effects of grape pomace and grape seed flours on cookie quality. Qual. Assur. Saf. Crop. Foods.

[62] Keriene I., Mankeviciene A., Bliznikas S., Jablonskyte-Rasce D., Maikštėnienė S., Česnulevičienė R. (2015). Biologically active phenolic compounds in buckwheat, oats and winter spelt wheat. Zemdirb. Agric..

[63] Rockenbach I., Rodrigues E., Gonzaga L.V., Genovese M.I., Gonçalves A.E., Fett R. (2011). Phenolic compounds content and antioxidant activity in pomace from selected red grapes (*Vitis vinifera* L. and *Vitis labrusca* L.) widely produced in Brazil. Food Chem..

[64] Iora S.R., Maciel G.M., Zielinski A.A., da Silva M.V., Pontes P.V.D.A., Haminiuk C.W., Granato D. (2015). Evaluation of the bioactive compounds and the antioxidant capacity of grape pomace. Int. J. Food Sci. Technol..

[65] Negro C., Aprile A., Luvisi A., De Bellis L., Miceli A. (2021). Antioxidant Activity and Polyphenols Characterization of Four Monovarietal Grape Pomaces from Salento (Apulia, Italy). Antioxidants.

[66] Cui W., Wang Y., Sun Z., Cui C., Li H., Luo K., Cheng A. (2023). Effects of steam explosion on phenolic compounds and dietary fiber of grape pomace. LWT-Food Sci. Technol..

[67] Putnik P., Bursać Kovačević D., Radojčin M., Dragović-Uzelac V. (2016). Influence of acidity and extraction time on the recovery of flavonoids from grape skin pomace optimized by response surface methodology. Chem. Biochem. Eng. Q..

[68] Ivanišová E., Ondrejovič M., Šilhár S. (2012). Antioxidant activity of milling fractions of selected cereals. Nova Biotechnol. Chim..

[69] Sumczynski D., Bubelova Z., Sneyd J., Erb-Weber S., Mlcek J. (2015). Total phenolics, flavonoids, antioxidant activity, crude fibre and digestibility in non-traditional wheat flakes and muesli. Food Chem..

[70] Abdel-Aal E.S.M., Rabalski I. (2008). Bioactive Compounds and their Antioxidant Capacity in Selected Primitive and Modern Wheat Species. Open Agric. J..

[71] Maner S., Sharma A.K., Banerjee K. (2017). Wheat flour replacement by wine grape pomace powder positively affects physical, functional and sensory properties of cookies. Proc. Natl. Acad. Sci. India Sect. B Biol. Sci..

[72] Sęczyk Ł., Świeca M., Gawlik-Dziki U. (2015). Changes of antioxidant potential of pasta fortified with parsley (Petroselinum Crispum mill.) leaves in the light of protein-phenolics interactions. Acta Sci. Pol. Technol. Aliment..

[73] Kruczek M., Gumul D., Korus A., Buksa K., Ziobro R. (2023). Phenolic Compounds and Antioxidant Status of Cookies Supplemented with Apple Pomace. Antioxidants.

[74] Ky I., Lorrain B., Kolbas N., Crozier A., Teissedre P.-L. (2014). Wine by-Products: Phenolic Characterization and Antioxidant Activity Evaluation of Grapes and Grape Pomaces from Six Different French Grape Varieties. Molecules.

[75] Žilić S., Kocadağlı T., Vančetović J., Gökmen V. (2016). Effects of baking conditions and dough formulations on phenolic compound stability, antioxidant capacity and color of cookies made from anthocyanin-rich corn flour. LWT.

[76] Francavilla A., Joye I.J. (2022). Anthocyanin Content of Crackers and Bread Made with Purple and Blue Wheat Varieties. Molecules.

[77] Santetti G.S., Dacoreggio M.V., Silva A.C.M., Biduski B., Bressiani J., Oro T., de Francisco A., Gutkoski L.C., Amboni R.D.M.C. (2021). Effect of yerba mate (Ilex paraguariensis) leaves on dough properties, antioxidant activity, and bread quality using whole wheat flour. J. Food Sci..

[78] Ozdal T., Capanoglu E., Altay F. (2013). A review on protein-phenolic interactions and associated changes. Food Res. Int..

[79] Chi C.H., Cho S.J. (2016). Improvement of bioactivity of soybean meal by solid-state fermentation with Bacillus amyloliquefaciens versus Lactobacillus spp. and Saccharomyces cerevisiae. LWT-Food Sci. Technol..

[80] Plustea L., Negrea M., Cocan I., Radulov I., Tulcan C., Berbecea A., Popescu I., Obistioiu D., Hotea I., Suster G. (2022). Lupin (*Lupinus* spp.)-Fortified Bread: A Sustainable, Nutritionally, Functionally, and Technologically Valuable Solution for Bakery. Foods.

[81] Dossa S., Negrea M., Cocan I., Berbecea A., Obistioiu D., Dragomir C., Alexa E., Rivis A. (2023). Nutritional, Physico-Chemical, Phytochemical, and Rheological Characteristics of Composite Flour Substituted by Baobab Pulp Flour (*Adansonia digitata* L.) for Bread Making. Foods.

[82] Lou W., Zhou K., Li B., Nataliya G. (2022). Rheological, pasting and sensory properties of biscuits supplemented with grape pomace powder. Food Sci. Technol..

[83] Sharma A.K., Dagadkhair R.A., Somkuwar R.G. (2018). Evaluation of grape pomace and quality of enriched cookies after standardizing baking conditions: Evaluation of grape pomace and quality of enriched cookies. J. AgriSearch.

[84] Azami S., Roufegari-Nejad L. (2019). The Effect of Red Grape Pomace Powder Replacement on Physical Characteristics and Acrylamide Content of Biscuit. Iranian J. Nutr. Sci. Food Technol..

[85] Ajila C.M., Leelavathi K., Prasada Rao U.J.S. (2008). Improvement of dietary fiber content and antioxidant properties in soft dough biscuits with the incorporation of mango peel powder. J. Cereal Sci..

